# The population genetics of speciation by cascade reinforcement

**DOI:** 10.1002/ece3.9773

**Published:** 2023-02-07

**Authors:** Carlie B. Anderson, Oscar Ospina, Peter Beerli, Alan R. Lemmon, Sarah E. Banker, Alyssa Bigelow Hassinger, Mysia Dye, Michelle L. Kortyna, Emily Moriarty Lemmon

**Affiliations:** ^1^ Department of Biological Science Florida State University Tallahassee Florida USA; ^2^ Department of Biostatistics and Bioinformatics Moffitt Cancer Center Tampa Florida USA; ^3^ Department of Scientific Computing Florida State University Tallahassee Florida USA; ^4^ Pfizer Clinical Pharmacogenomics Group Groton Connecticut USA; ^5^ Varigen Biosciences Middleton Wisconsin USA

**Keywords:** cascade reinforcement, character displacement, hybridization, population genetics, speciation

## Abstract

Species interactions drive diverse evolutionary outcomes. Speciation by cascade reinforcement represents one example of how species interactions can contribute to the proliferation of species. This process occurs when the divergence of mating traits in response to selection against interspecific hybridization incidentally leads to reproductive isolation among populations of the same species. Here, we investigated the population genetic outcomes of cascade reinforcement in North American chorus frogs (Hylidae: *Pseudacris*). Specifically, we estimated the frequency of hybridization among three taxa, assessed genetic structure within the focal species, *P*. *feriarum*, and ascertained the directionality of gene flow within *P*. *feriarum* across replicated contact zones via coalescent modeling. Through field observations and preliminary experimental crosses, we assessed whether hybridization is possible under natural and laboratory conditions. We found that hybridization occurs among *P*. *feriarum* and two conspecifics at a low rate in multiple contact zones, and that gene flow within the former species is unidirectional from allopatry into sympatry with these other species in three of four contact zones studied. We found evidence of substantial genetic structuring within *P*. *feriarum* including a divergent western allopatric cluster, a behaviorally‐distinct sympatric South Carolina cluster, and several genetically‐overlapping clusters from the remainder of the distribution. Furthermore, we found sub‐structuring between reinforced and nonreinforced populations in the two most intensely‐sampled contact zones. Our literature review indicated that *P*. *feriarum* hybridizes with at least five heterospecifics at the periphery of its range providing a mechanism for further intraspecific diversification. This work strengthens the evidence for cascade reinforcement in this clade, revealing the geographic and genetic landscape upon which this process can contribute to the proliferation of species.

## Graphical abstract

Here, we investigated the population genetic outcomes of cascade reinforcement, a powerful mode of diversification, to provide the first insight into species‐wide patterns of population genetic differentiation and gene flow that lend support to theoretical predictions. This work advances our understanding of how complex species interactions contribute to the proliferation of species and the potential for rapid diversification in our geographically‐ and genetically‐unique study system.

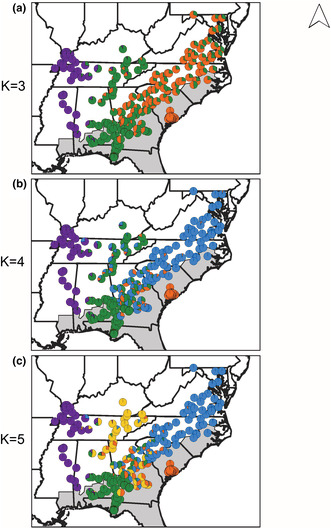

## 1 INTRODUCTION

Species interactions are a powerful evolutionary force contributing to the generation and maintenance of biodiversity (Mayr, 1963; see Rabosky, 2013 for review). Where related species coexist, interspecific interactions often result in reduced fitness in one or both interacting taxa, driving the evolution of novel biological solutions to this common problem. When these solutions result in differential selection of mating traits among populations, species interactions may shape evolutionary trajectories (Hoskin & Higgie, 2010). The presence of closely‐related heterospecifics is one type of species interaction that may drive an increase in assortative mating, if interspecific matings result in less fit hybrid offspring (Arntzen & Wallis, 1991; Cruzan & Arnold, 1993; Harrison, 1986; Naisbit et al., 2001; Sætre et al., 1997). Species interactions may drive even more dramatic evolutionary outcomes, like hyperdiverse adaptive radiations that result from resource competition (Levis et al., 2017; MacLean et al., 2004; Rüber et al., 1999; Schluter, 1994; Seehausen, 2006). The evolutionary impacts of species interactions remain a novel research area for ecologists and evolutionary biologists alike.

Reinforcement, the evolution of premating isolating barriers in response to selection against hybridization (Dobzhansky, 1940; Howard, 1993), represents one type of species interaction that can promote diversification both between and within species. One characteristic outcome of reinforcement is the divergence of premating signaling traits between interacting taxa, termed reproductive character displacement (RCD; Howard, 1993), driven by selection to reduce interspecific matings. Theoretical work has demonstrated that divergence of these traits can also have the indirect effect of reducing matings between allopatric and sympatric populations of the same species (Calabrese & Pfennig, 2020; Hoskin & Higgie, 2010; McPeek & Gavrilets, 2006; Ortiz‐Barrientos et al., 2009; Pfennig & Pfennig, 2009; Pfennig & Ryan, 2006). Empirical evidence for this mode of diversification, termed cascade reinforcement (Ortiz‐Barrientos et al., 2009), is now well‐documented in a variety of animal taxa (e.g., in invertebrates: Dyer et al., 2014; Humphreys et al., 2016; Jaenike et al., 2006; Nosil et al., 2003; Porretta & Urbanelli, 2012; in fish: Kozak et al., 2015; Moran & Fuller, 2018; and in amphibians: Lemmon, 2009; Pfennig & Rice, 2014; Rice & Pfennig, 2010).

Contact zones are a natural laboratory for the study of speciation by cascade reinforcement. Analysis of genetic variation across a contact zone allows for testing hypotheses related to patterns of gene flow, the relative strength of selection, and the genetic architecture of divergent characters (Barton & Hewitt, 1985). Empirical studies of cascade reinforcement and RCD have primarily compared mate preferences (Humphreys et al., 2016; Jaenike et al., 2006; Kozak et al., 2015; Moran & Fuller, 2018; Nosil et al., 2002) or fertility of hybrid offspring (Comeault et al., 2016; Ostevik et al., 2020) across sympatric and allopatric populations. The literature on both cascade reinforcement and reinforcement in the classical sense, however, has largely neglected to test for population genetic patterns predicted to be associated with these processes (exceptions in reinforcement: Hopkins et al., 2012; Roda et al., 2017; in cascade reinforcement: Bewick & Dyer, 2014; Pfennig & Rice, 2014). Testing the population genetic predictions of cascade reinforcement theory is challenging due to the difficulty of distinguishing the relative contributions of this process and environmental variation on phenotypic and genetic differentiation (i.e., ruling out ecological divergence: Doebeli & Dieckmann, 2000; Gavrilets, 2004; Rundle & Nosil, 2005; Schluter, 2001).

North American chorus frogs (Hylidae: *Pseudacris*) represent an ideal model to assess the population genetic predictions of cascade reinforcement. The Upland chorus frog (*Pseudacris feriarum*) diverged from its congener, *P*. *nigrita*, ~8 mya (Lemmon, Lemmon, Collins, et al., 2007). The former species has since expanded its range in the last 10,000 years (Lemmon & Lemmon, 2008), invading the range of *P*. *nigrita* by following river drainages in the Coastal Plain of the southeastern U.S. in at least five, phylogenetically‐independent instances (Banker et al., 2020). Interspecific hybrids experience reduced fitness relative to the parental species, and this cost of hybridization has led to RCD of male advertisement calls and female preferences for these signals (Lemmon, 2009; Lemmon & Lemmon, 2010) that cannot be attributed to environmental selection (Malone et al., 2014). Reinforcement in sympatry with *P*. *nigrita* has indirectly strengthened reproductive isolation among populations of *P*. *feriarum* (Lemmon, E. M., Ospina, O. E., Kortyna, M., Hassinger, A. B., Dye, M., Holland, S., Booker, W., Cherry, J. R., & Lemmon, A.R., unpublished data; Lemmon, 2009; Lemmon & Lemmon, 2010) as predicted from cascade reinforcement theory (McPeek & Gavrilets, 2006; Ortiz‐Barrientos et al., 2009; Pfennig & Ryan, 2006). Moreover, also consistent with the latter process, genetic variation within *P*. *feriarum* is not explained by isolation‐by‐distance alone (Banker et al., 2020).

Despite the behavioral evidence to support cascade reinforcement in *P*. *feriarum*, we lack clear tests of the population genetic patterns predicted to result from this process. Contrary to the prediction [PREDICTION 1] that reinforcement should decrease rates of hybridization (Blair, 1974; Coyne & Orr, 2004; Dobzhansky, 1940; Jones, 1973; Pfennig, 2003; but see Butlin, 1987), Lemmon and Juenger (2017) estimated the extent of hybridization between *P*. *feriarum* and *P*. *nigrita* to be high (32% F1 hybrids in one sympatric population). These estimates of gene flow had substantial error margins, however, and a later estimate of hybridization in one contact zone suggested substantially lower levels of admixture (Banker et al., 2020). Here, we revisit these estimates of hybridization among three interacting species, one of which demonstrates phenotypic patterns indicative of cascade reinforcement (focal species *P*. *feriarum*; Lemmon, 2009).

Prior studies have predicted [PREDICTION 2] that cascade reinforcement contributes to decreased gene flow between sympatric and allopatric populations (Pfennig & Pfennig, 2009; Pfennig & Rice, 2007, 2014; Servedio, 2004). Lemmon and Juenger (2017) found evidence for increased genetic differentiation among sympatric and allopatric populations based on estimates from a small number of microsatellite markers. With a larger genomic data set, we aim to generate a more complete description of genetic structure within the focal species, *P*. *feriarum*, to qualitatively assess genetic divergence between reinforced and nonreinforced populations.

Theory also predicts [PREDICTION 3] that bidirectional gene flow is most conducive to reinforcement (Servedio & Kirkpatrick, 1997). Banker et al. (2020) found, however, some evidence in *P*. *feriarum* for directional gene flow from allopatry into sympatry. We build upon these findings to determine the degree of parallel directional gene flow within the focal species across multiple replicate contact zones. The directionality of gene flow in a contact zone can offer insight into the strength of selection against matings between populations and reproductive isolation within the focal species (Coyne & Orr, 2004; Kelly & Noor, 1996; Kirkpatrick & Servedio, 1999; Servedio & Kirkpatrick, 1997).

**FIGURE 1:**
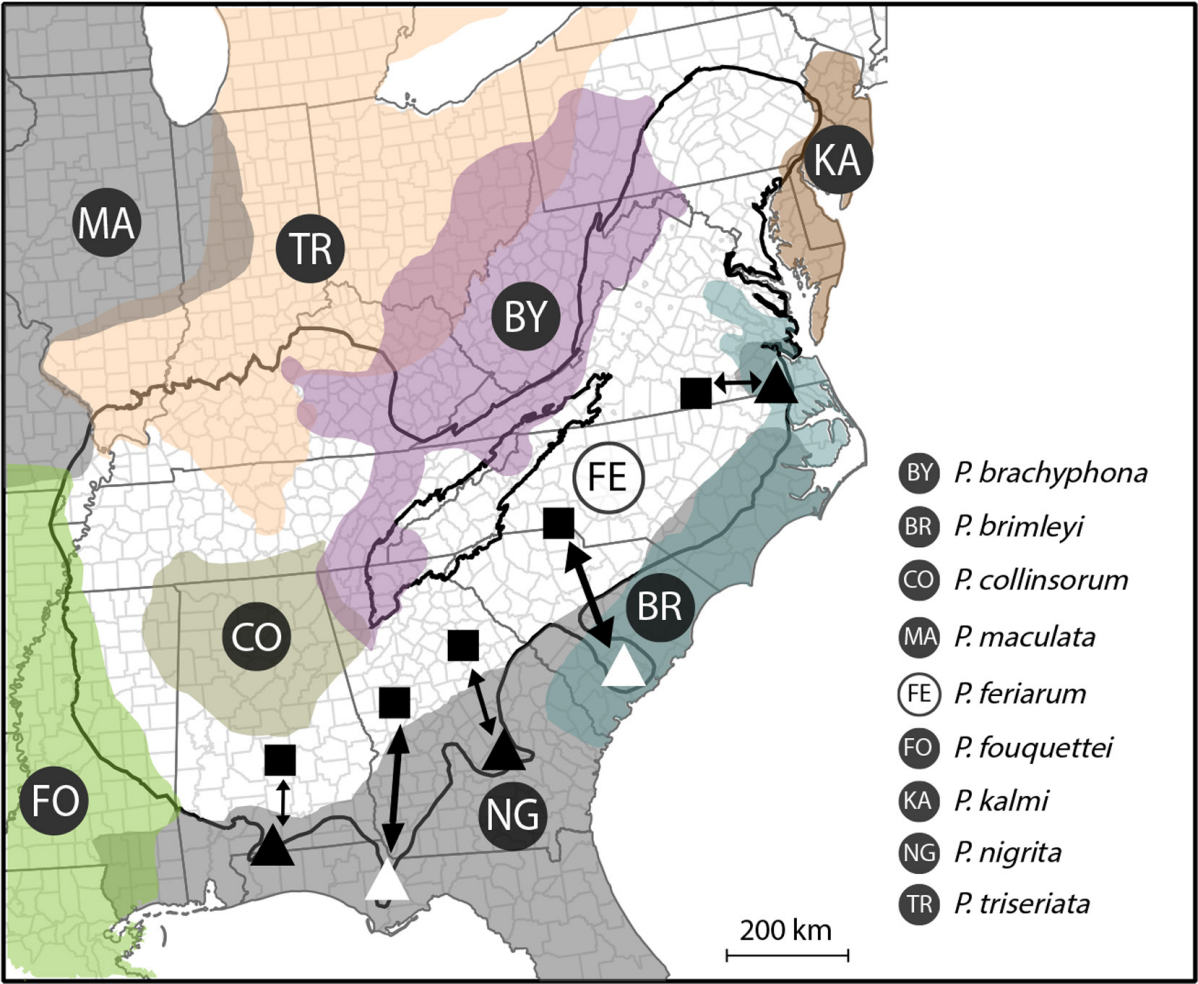
Representative schematic of the study system and population genetic analyses conducted. The ranges of *Pseudacris feriarum* and eight congeneric taxa with which the focal species experiences some degree of range overlap are shown in transparent overlays. The five heavily‐sampled contact zones between *P*. *feriarum* and at least one congener in this study are represented by pairs of *P*. *feriarum* populations that are sympatric (triangle) and allopatric (square) with respect to either *P*. *nigrita* or both *P*. *nigrita* and *P*. *brimleyi*. The two sympatric populations included in our analyses of interspecific hybridization are symbolized by white triangles, and the remaining three sympatric populations are symbolized by black triangles. Bidirectional arrows represent our estimation of the directionality of gene flow between allopatric and sympatric pairs of *P*. *feriarum* populations.

A final prediction [PREDICTION 4] is that interactions among multiple (more than two) species can increase the power of cascade reinforcement to accelerate diversification and the evolution of reproductive isolation (Calabrese & Pfennig, 2020; McPeek & Gavrilets, 2006; Pfennig & Ryan, 2007). To test this hypothesis, we assess the presence of hybridization among different heterospecifics. Hybrid offspring between *P*. *feriarum* and *P*. *nigrita* have been found in sympatry (Lemmon, 2009). Furthermore, hybrids with other closely‐related species have occasionally been observed along the periphery of the range of *P*. *feriarum* (e.g., *P*. *brachyphona* and others; Lemmon, Lemmon, & Cannatella, 2007; Figure 1). We assessed the potential for multiple species to accelerate cascade reinforcement by summarizing the evidence for hybridization of *P*. *feriarum* with multiple congeners.

The goal of this study is to assess population genetic patterns that parallel ongoing cascade reinforcement within *P*. *feriarum* (Figure 1). We specifically ask: (1) How common is hybridization among three‐species diverging via this process—*P*. *feriarum*, *P*. *nigrita*, and *P*. *brimleyi*?, (2) What degree of genetic structure is present within the focal species *P*. *feriarum*?, (3) What is the directionality of gene flow across replicated hybrid zones between *P*. *feriarum* and *P*. *nigrita*?, and (4) Does the focal species *P*. *feriarum* hybridize with other congeneric taxa at the periphery of its range? We build upon Lemmon and Juenger's (2017) microsatellite analyses and Banker et al.'s (2020) regional estimate of admixture by using a more powerful set of genome‐wide nuclear markers across multiple contact zones. We combine these population genetic analyses with laboratory crosses and field observations, as well as a literature review of hybridization across *Pseudacris* species. This study is the first to shed light on the species‐wide population genetic patterns that can arise during speciation via cascade reinforcement.

## 2 METHODS

### 2.1 Sampling

A total of 765 chorus frogs were sampled throughout the ranges of *Pseudacris* species with overlapping range boundaries. In all, 567 putative *P*. *feriarum*, 125 *P*. *nigrita*, and 73 *P*. *brimleyi* were sampled in allopatry and sympatry, across five replicate contact zones between species (Alabama, Florida, Georgia, South Carolina, and Virginia; Banker et al., 2020). Particularly intense sampling was conducted in two of these zones, Florida and South Carolina, where high levels of call divergence resulting from reinforcement have been observed (Lemmon, 2009; Lemmon & Lemmon, 2010). In the Florida contact zone, 116 *P*. *feriarum* and 31 *P*. *nigrita* were sampled in sympatry in addition to 91 *P*. *feriarum* and 47 *P*. *nigrita* pure‐species controls from nearby allopatry. In the South Carolina sympatric zone, 214 *P*. *feriarum*, 45 *P*. *nigrita*, and 49 *P*. *brimleyi* were sampled in sympatry in addition to 66 *P*. *feriarum*, 47 *P*. *nigrita*, and 24 *P*. *brimleyi* pure‐species controls from nearby allopatry. Note that some of the same allopatric samples were included in the analyses of these two contact zones, which is why the sum of these numbers exceeds the total number of frogs in this study (taxa included in each analysis listed in Table S1). Seven lab‐created *P*. *feriarum*–*P*. *brimleyi* F1 hybrids were also included for comparison to field‐caught samples. Therefore, a total of 772 individual genetic samples were included in this study. Appropriate state scientific collecting permits and ACUC protocol approvals were obtained prior to specimen collection. Tissues were frozen in liquid nitrogen or preserved in tissue buffer or 95% ethanol and stored at −80°C. Specimen vouchers were deposited into the Texas Natural History Collection or the University of Florida Museum of Natural History (Table S1).

### 2.2 Data generation and assembly

Samples were genotyped using Anchored Hybrid Enrichment (AHE; Lemmon et al., 2012) at Florida State University's Center for Anchored Phylogenomics (www.anchoredphylogeny.com). AHE is a hybrid enrichment (sequence capture) approach for recovering hundreds of unique, genome‐wide orthologous loci for resolving evolutionary relationships within nonmodel systems on both shallow and deep scales (Lemmon et al., 2012; Lemmon & Lemmon, 2013). Genomic DNA was extracted from samples using OMEGA Bio‐tek E.Z.N.A Tissue DNA kit. After extraction, genomic DNA was sonicated to a fragment size of ~300–800 bp using a Covaris E220 Focused‐ultrasonicator with Covaris microTUBES. Library preparation and indexing were performed on a Beckman‐Coulter Biomek FXp liquid‐handling robot following Meyer and Kircher (2010) but with size selection after blunt‐end repair using SPRI select beads (Beckman‐Coulter Inc.; 0.9× ratio of bead to sample volume). Indexed samples were pooled at equal quantities (with 16 samples per pool) and enriched using the Anchored Hybrid Enrichment *Pseudacris* v1 kit (Agilent Technologies Custom SureSelect XT kit) described by Banker et al. (2020), which was developed using sequences from *P*. *feriarum* and *P*. *nigrita*. Enriched library pools were pooled in equal quantities and sequenced on PE150 Illumina HiSeq 2500 lanes at the Translational Science Laboratory in the College of Medicine at Florida State University. Prior to demultiplexing with no mismatches tolerated, low‐quality reads were removed using the Cassava high chastity filter setting.

### 2.3 Read quality control and processing

Read processing and assembly followed the methods developed by Hamilton et al. (2016), and first applied to *Pseudacris* by Banker et al. (2020). In brief, overlapping reads were merged following Rokyta et al. (2012) then assembled using the *P*. *feriarum* and *P*. *nigrita* probe‐design reference sequences. Assembly clusters with read coverage above the 5th percentile were retained (91 reads on average). Orthology was assessed using pairwise sequence distances as described by Hamilton et al. (2016). Alleles were phased assuming diploidy following Pyron et al. (2016) then aligned using MAFFT v7.023b (Katoh & Standley, 2013), with ‐‐genafpair and ‐‐maxiterate 1000 flags utilized. The alignment for each locus was then trimmed/masked using the script developed by Hamilton et al. (2016) with settings (MINGOOD = 16, MISSINGALLOWED = 50%). Sequences containing an excessive number of heterozygosities (more than 10) were removed, as they suggest recent gene duplication. Finally, visual inspection of each masked alignment was carried out in Geneious version 7 (www.geneious.com; Kearse et al., 2012), after which regions of sequences identified as obviously misaligned or paralogous were removed.

### 2.4 Admixture estimation

SNPs were identified to generate three data sets: (1) a Range‐wide data set of *n* = 560 *P*. *feriarum* individuals from throughout the species' range, (2) a Florida data set of *n* = 285 individuals from the Florida contact zone and adjacent areas, identified in the field as *P*. *feriarum* (*n* = 207), and *P*. *nigrita* (*n* = 78), and (3) a South Carolina data set of *n* = 452 individuals from the South Carolina contact zone and adjacent areas, identified in the field as *P*. *feriarum* (*n* = 280), *P*. *nigrita* (*n* = 92), *P*. *brimleyi* (*n* = 73), and captive‐bred *P*. *feriarum*–*P*. *brimleyi* F1 hybrid (*n* = 7) individuals, for model validation (Table S1). After isolating the target individuals for each of these data sets, SNPs were extracted using a custom script that: (1) identifies stretches of seven consecutive sites each represented by at least 50% of taxa, (2) verifies that the central site in each stretch has a minor allele count of at least three (Linck & Battey, 2019) to remove invariable sites and singletons, (3) verifies that the three flanking sites on each side of the central site each have 80% identity or greater, and (4) retains for each locus only the central‐most verified SNP (to satisfy the independent site assumption). This procedure produced *n* = 613, *n* = 611, and *n* = 603 SNPs for the Range‐wide, Florida, and South Carolina data sets, respectively.

To assess population structure and evidence for admixture, data were analyzed using fastSTRUCTURE as integrated into Structure_threader (Pina‐Martins et al., 2017; Pritchard et al., 2000; Raj et al., 2014) under the basic admixture model, convergence criterion 1 × 10^−6^, and 100 cross‐validation repetitions; default settings were employed for other parameters. Analyses were conducted assuming K = 1 to K = 8. The optimal K‐value was identified using the “fastChooseK.py” script implemented in Structure_threader. This approach generates a range of most likely K's bounded by the result of a maximum likelihood estimation and the result of a model complexity estimation (Raj et al., 2014). fastSTRUCTURE analyses were run on all three data sets using the high‐performance computing cluster at Florida State University.

### 2.5 Hybrid index estimation

To determine the hybrid index of putative hybrids, GenoDive v3.02 (Meirmans, 2020) analyses were performed on three additional data sets, each containing one SNP per locus, using subsets of individuals from the fastSTRUCTURE analyses above: (4) Florida *P*. *feriarum* and *P*. *nigrita* samples (*n* = 147), (5) South Carolina *P*. *feriarum* and *P*. *nigrita* samples (*n* = 259), and (6) South Carolina *P*. *feriarum* and *P*. *brimleyi* samples (*n* = 269; Table S1). The latter analysis included seven laboratory‐raised hybrids between *P*. *feriarum* and *P*. *brimleyi* for comparison of hybrid indices to putative natural hybrids. Analyses were performed as described in Lemmon and Juenger (2017) and Banker et al. (2020), setting allopatric *P*. *feriarum* as the reference species and allopatric individuals of the other species as the alternative. We identified an individual as admixed if the upper and lower bounds of the hybrid index 95% confidence intervals did not include 0 or 1 (following Lemmon & Juenger, 2017).

### 2.6 Intraspecific genetic structure

Discriminant analysis of principal component (DAPC; Jombart et al., 2010) was employed to identify intraspecific genetic clusters within the Range‐wide *P*. *feriarum* data set (613 SNPs, one SNP per locus, *n* = 560 individuals). DAPC is an unsupervised‐supervised clustering method that relies on the selection of different sets of alleles that best describe the genetic similarities among samples. The linear allele combinations are known as Discriminant Functions (DFs) that maximize the genetic variation among clusters while minimizing the diversity within clusters. The data set was formatted into a Structure file with PGDSpider v2.1.1.5 in order to analyze it in the R package “adegenet” (Jombart, 2008). The range of most likely clusters in our Range‐wide *P*. *feriarum* data set was inferred by using the “find.clusters” function. The function provides a Bayesian Information Criterion (BIC) as a metric to select the most likely number of clusters, which we assessed from K = 1 to K = 20 via 1 × 10^6^ replicates. For each of the most likely Ks, 100 cross‐validations were performed (function “xvalDapc”) to obtain the minimum number of principal components (PC) that best described the variation within the proposed clusters while reducing the chance of model overfitting. Finally, DFs were generated for each likely K in the data set (function “dapc”). Each individual was assigned to a cluster by selecting the two most informative DFs.

### 2.7 Genetic structure summary statistics

To determine the level of divergence between populations and diversity within populations, several standard population genetic statistics were estimated using the packages “SambaR” (Jong et al., 2021), “adegenet” (Jombart, 2008), and “StAMPP‐1.6.3” (Pembleton et al., 2013). For these analyses, the Range‐wide *P*. *feriarum* data set was subset into ten groups of between 6 and 22 individuals, matching the population designations in the *Directionality of Gene Flow* analyses below (613 SNPs, one SNP per locus, *n* = 132 individuals) using the “keep” function in VCFtools (Danecek, et al., 2011). The R package “vcfR” (Knaus & Grünwald, 2017) was then used to convert the file to the genind and genlight formats for downstream analyses and the addition of population designations. Prior to analyses, the data were filtered using the function “filterdata” of the R package “SambaR”, with indmiss = 0.2, snpmiss = 0.1, min_mac = 2, dohefilter = TRUE, and min_spacing = 500. After filtering 126 out of 132 individuals (5–21 per population) were retained (Table S2). To determine the level of divergence between populations, the following pairwise statistics were calculated and visualized using the main function “calcdistance” in the R package “SambaR” (Jong et al., 2021): Nei's genetic distance (Nei, 1972); a genome‐wide estimation of Weir and Cockerham (1984)'s F_ST_; and mean D_xy_. The “calcdistance” function calls on the functions “stamppNeisD” and “stamppFst” of the R package “StAMPP‐1.6.3” (Pembleton et al., 2013). Additionally, a test for significant population differentiation by comparison of F_ST_ values was conducted using the “stamppFst” function in the R package “StAMPP‐1.6.3” (Pembleton et al., 2013). To determine the level of diversity within and between populations, mean nucleotide diversity (*π*) and Tajima's *D* were calculated and visualized using the main function “calcdiversity” in the package “SambaR” (Jong et al., 2021).

### 2.8 Directionality of gene flow

To assess the direction and magnitude of migration across five replicate contact zones within the broader hybrid zone, MIGRATE‐n v4.4 (Beerli et al., 2022) was used to analyze Anchored Hybrid Enrichment (AHE) sequence data of *P*. *feriarum* from a previous study (Banker et al., 2020). MIGRATE‐n is a Bayesian coalescent‐based algorithm that estimates the mutation‐scaled population sizes (*θ*) and mutation‐scaled gene flow rates (*M*) among populations (Beerli et al., 2019); *θ* is 4 times the effective population size times the mutation rate per site and generation, *M* is a ratio between the immigration rate and the mutation rate, we assume that the mutation rate is the same among all species and we use *θ* and *M* instead of absolute numbers. AHE sequence data from Banker et al. (2020) were combined with newly acquired AHE loci for an additional number of *P*. *feriarum*, resulting in a data set of 402 sequenced individuals. From the 758 AHE loci, 100 loci were selected that showed no poorly aligned regions and no obvious paralogy (Banker et al., 2020). We selected 100 loci for computational reasons; however, to our knowledge, there is no formal framework for assessing the number of loci necessary for model testing within MIGRATE‐n. The allopatric populations of *P*. *feriarum* occur in the geographic continuum (Lemmon & Juenger, 2017), thus to define populations to perform model testing with MIGRATE‐n, circles of diameter 150 km were drawn in QGIS v2.14.19 (QGIS Development Team, 2017) that collected as many individuals as possible from each of the previously defined DAPC clusters. The same procedure was repeated for the sympatric populations. As a result, 10 populations were defined (five allopatric and five sympatric; Figure S1), representing the five incursions into sympatry previously described for this species (Banker et al., 2020). In two instances (South Carolina “orange” and Florida “green” clusters), the DAPC clusters were composed only of sympatric individuals. To generate an allopatric counterpart for those sympatric populations, allopatric individuals from the Virginia and Georgia incursions, respectively, were selected. For each of the five incursions into sympatry, three different models aiming to measure the direction and magnitude of gene flow into sympatry were tested: (A) migration only from allopatry to sympatry, (B) migration only from sympatry to allopatry, and (C) migration in both directions (Figure S1). Before running the analyses, at most 20 individuals within each circle were randomly subsampled. Three simultaneous MCMC chains were run for each model, with 50,000 steps sampled every 50 steps. Uniform priors were selected for migration rates and population sizes. Other criteria were left as default and parameter files (a.k.a. parmfiles) for the analyses are available in the Dryad data depository. Analyses were conducted using the resources at FSU's Research Computing Center.

### 2.9 Laboratory hybrid crosses

Evidence for natural hybridization in the field has been demonstrated previously between *P*. *feriarum* and *P*. *nigrita* (Banker et al., 2020; Lemmon & Juenger, 2017) but not between *P*. *feriarum* and *P*. *brimleyi*. By generating crosses between the latter species pair, two questions were addressed: (1) Can the species produce viable hybrid offspring?, (2) Do hybrid crosses have lower hatching success (#tadpoles hatched/#eggs produced × 100) than pure parental species crosses? For this experiment, wild‐caught frogs were crossed under laboratory conditions described in Lemmon and Lemmon (2010). Three pure *P*. *brimleyi* crosses, five pure *P*. *feriarum* crosses, and six hybrid crosses were generated (two with *P*. *brimleyi* as the male and four with *P*. *feriarum* as the male). Parents were collected from sympatry in Colleton and Dorchester counties, South Carolina (Table S3). Mating and hatching success were observed and quantified for each cross. Tadpole hatching success was compared between hybrid and pure species crosses via a randomization test in R (R Core Team, 2020). The parents and a subset of tadpole offspring from these laboratory crosses were also genotyped to verify the power of our genetic markers for identifying wild‐caught hybrids (Table S1).

### 2.10 Field observations of heterospecific interactions

Field surveys were conducted in 2004, 2005, and 2013 to determine the extent of natural interaction among *P*. *feriarum*, *P*. *nigrita*, and *P*. *brimleyi* at breeding sites. These surveys were performed opportunistically and did not follow preplanned transects. Species presence within each site was assessed by making acoustic surveys of male mating calls and by observing the morphology of captured specimens. Mixed species choruses and any heterospecific amplexed pairs were documented at each locality.

### 2.11 Hybridization among the Trilling *Pseudacris* species

Information pertaining to hybridization among *P*. *feriarum* and all other members of the Trilling Chorus Frog Clade (Moriarty & Cannatella, 2004) was gathered from published genetic studies (Cambridge, 2018; Engebretsen et al., 2016; Gartside, 1980; Lemmon & Juenger, 2017; Lemmon, Lemmon, & Cannatella, 2007; Moriarty & Cannatella, 2004; Ospina et al., 2020) and unpublished genetic data sets (Dye, M., unpublished data; Lemmon, E. M., Ospina, O. E., Kortyna, M., Hassinger, A. B., Dye, M., Holland, S., Booker, W., Cherry, J. R., & Lemmon, A.R., unpublished data). Hybridization between a species pair was confirmed if: (1) an individual showed a mismatch between its mitochondrial DNA haplotype and either its acoustic or morphological species identity or (2) if analyses of an individual's nuclear DNA sequence data supported its placement as an F1 hybrid or F1 hybrid backcross.

## 3 RESULTS

### 3.1 Data recovery

An average of 6.6 million read pairs was obtained per sample, totaling 158 trillion nucleotide bases. The assemblies recovered an average of approximately 1400 loci per individual, with an average consensus sequence length of 1161 bp per locus. The combined, trimmed, alignment was comprised of 756 loci and 703,210 sites (61,538 informative), with only 2.2% ambiguous/missing characters. The average locus contained 930 sites.

### 3.2 Admixture and hybrid index estimation

Natural hybridization was detected among *P*. *feriarum*, *P*. *nigrita*, and *P*. *brimleyi*. Analyses using fastSTRUCTURE identified low levels of admixture between *P*. *feriarum* and *P*. *nigrita* in all three data sets, and between *P*. *feriarum* and *P*. *brimleyi* in the South Carolina data set (Figures S2–S4; Tables S4–S23).

**FIGURE 2:**
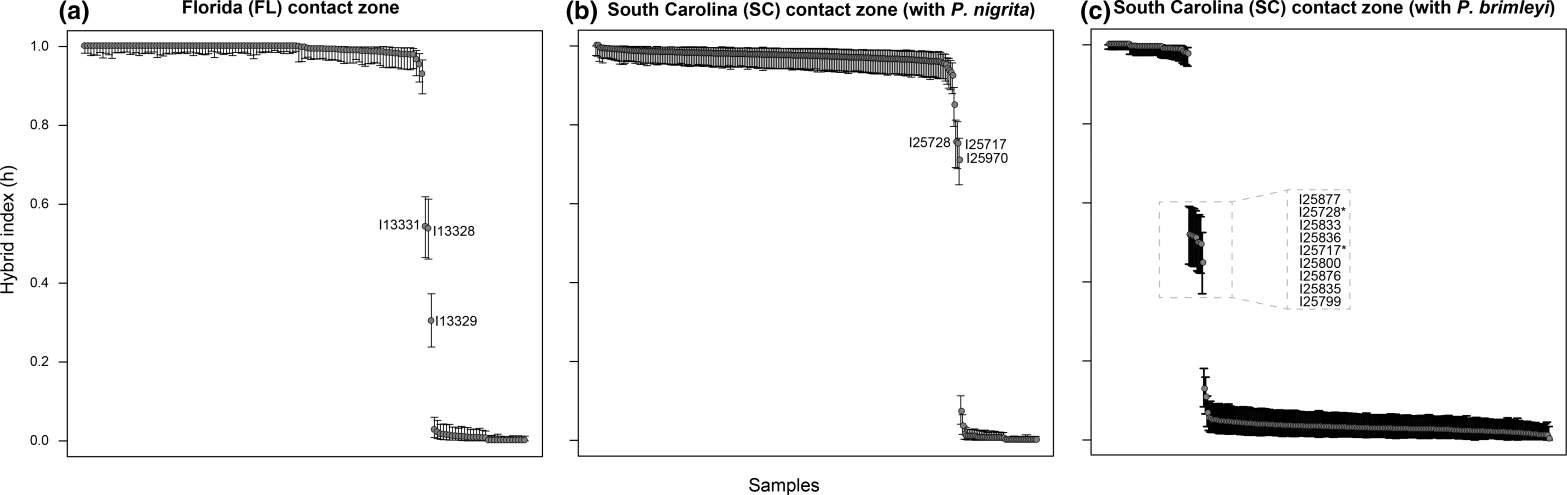
Hybrid indexes (h) for individuals collected at two contact zones between species. In all cases, values near 1 or 0 indicate “pure” genotypes of one of the species involved in the analysis. Intermediate values indicate admixed individuals (filled circles) with different genetic proportions of each species. (a) *P*. *feriarum* (h ~ 1) vs. *P*. *nigrita* (h ~ 0) in the Apalachicola River river drainage of Florida (FL). (b) *P*. *feriarum* (h ~ 1) vs. *P*. *nigrita* (h ~ 0) in Edisto‐Santee river drainage of South Carolina (SC). (c) *P*. *brimleyi* (h ~ 1) vs. *P*. *feriarum* (h ~ 0) in the Edisto‐Santee river drainage of South Carolina (SC). In panel (c), asterisks mark two natural hybrids captured in the field; the remaining seven hybrids were lab‐generated. Error bars represent confidence intervals for each hybrid index estimate (Tables S24–S26).

Several types of hybrids were identified in regions of sympatry. Admixed individuals between *P*. *feriarum* and *P*. *nigrita* were detected at 2% (three admixed individuals out of 147 sampled) in Florida and 1.2% (three admixed individuals out of 259 sampled) in South Carolina but estimated at 0.74% in South Carolina between *P*. *feriarum* and *P*. *brimleyi* (two admixed individuals out of 269 sampled; Tables S24–S26). Admixed individuals were identified if the hybrid index 95% confidence intervals did not include 0 or 1 (Tables S24–S26). GenoDive analyses indicated that in Florida, putative F1 hybrids (*n* = 2) between *P*. *feriarum* and *P*. *nigrita* were present, as well as one F1 hybrid backcross to *P*. *feriarum* (Figure 2). In South Carolina, F1 hybrids (*n* = 2) were found between *P*. *feriarum* and *P*. *brimleyi* in addition to F1 *P*. *feriarum* and *P*. *nigrita* hybrid backcrosses to *P*. *feriarum* (*n* = 3; Figure 2). The lab‐generated hybrid tadpoles and their parents genotyped from the crossing experiment were classified by GenoDive in a manner consistent with their pedigree, lending credence to the assumption that wild‐caught frogs were accurately assigned to genetic clusters (Tables S24–S26).

### 3.3 Genetic structure within *P*. *feriarum*

**FIGURE 3:**
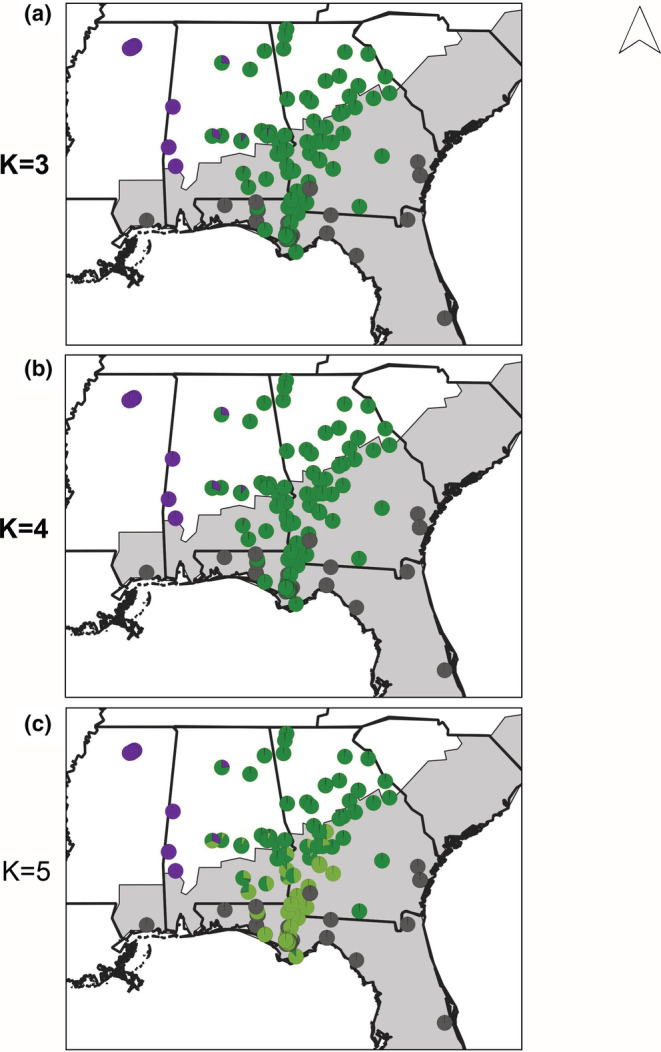
Geographic locations and admixture coefficients within the Florida (FL) contact zone. Analyses were conducted in fastSTRUCTURE with *P*. *feriarum* and *P*. *nigrita* for the most likely cluster configurations (bolded, K = 3 and K = 4 were best‐supported; Figure S2; Table S27). Gray‐shaded area of the map represents the range of *P*. *nigrita*. For K = 3 (a) and K = 4 (b), dark gray indicates sympatric and allopatric *P*. *nigrita*, green indicates sympatric and allopatric *P*. *feriarum*, and purple indicates western allopatric *P*. *feriarum*. Three colors are shown at K = 4 (b) since the fourth genetic cluster had very low admixture coefficients that could not be visualized (Table S13). At K = 5 (c), dark green indicates an additional allopatric *P*. *feriarum* cluster and lime green indicates a sympatric *P*. *feriarum* cluster. Four colors are shown at K = 4 (c) since the fifth genetic cluster had very low admixture coefficients that could not be visualized (Table S14).

In Florida and adjacent areas (Florida data set), both of the two best models of genetic structure (K = 3 and K = 4; Table S27) point to a deep divergence within *P*. *feriarum*, which reveals a cryptic, geographically‐localized group, and a shallower divergence between sympatric and allopatric populations of the focal species. Using fastSTRUCTURE, at K = 3, sympatric and allopatric *P*. *nigrita* formed one cluster (dark gray), sympatric and most allopatric *P*. *feriarum* formed a second cluster (green), and western allopatric *P*. *feriarum* formed a third (purple). The split of *P*. *feriarum* into two groups at K = 3 corresponds to a deep divergence at the base of the *P*. *feriarum* phylogeny between the western allopatric cluster and the remainder of the species. The other best model, K = 4, introduced an additional cluster supported at very low percentage (~0.024%) in all individuals, and thus did not contribute further to explaining population structure (Figure 3; Figure S2; Tables S11–S17). At higher K values (5, 6, and 8), Florida *P*. *feriarum* was further split into allopatric (dark green) and sympatric (lime green) clusters (Figure 3).

**FIGURE 4:**
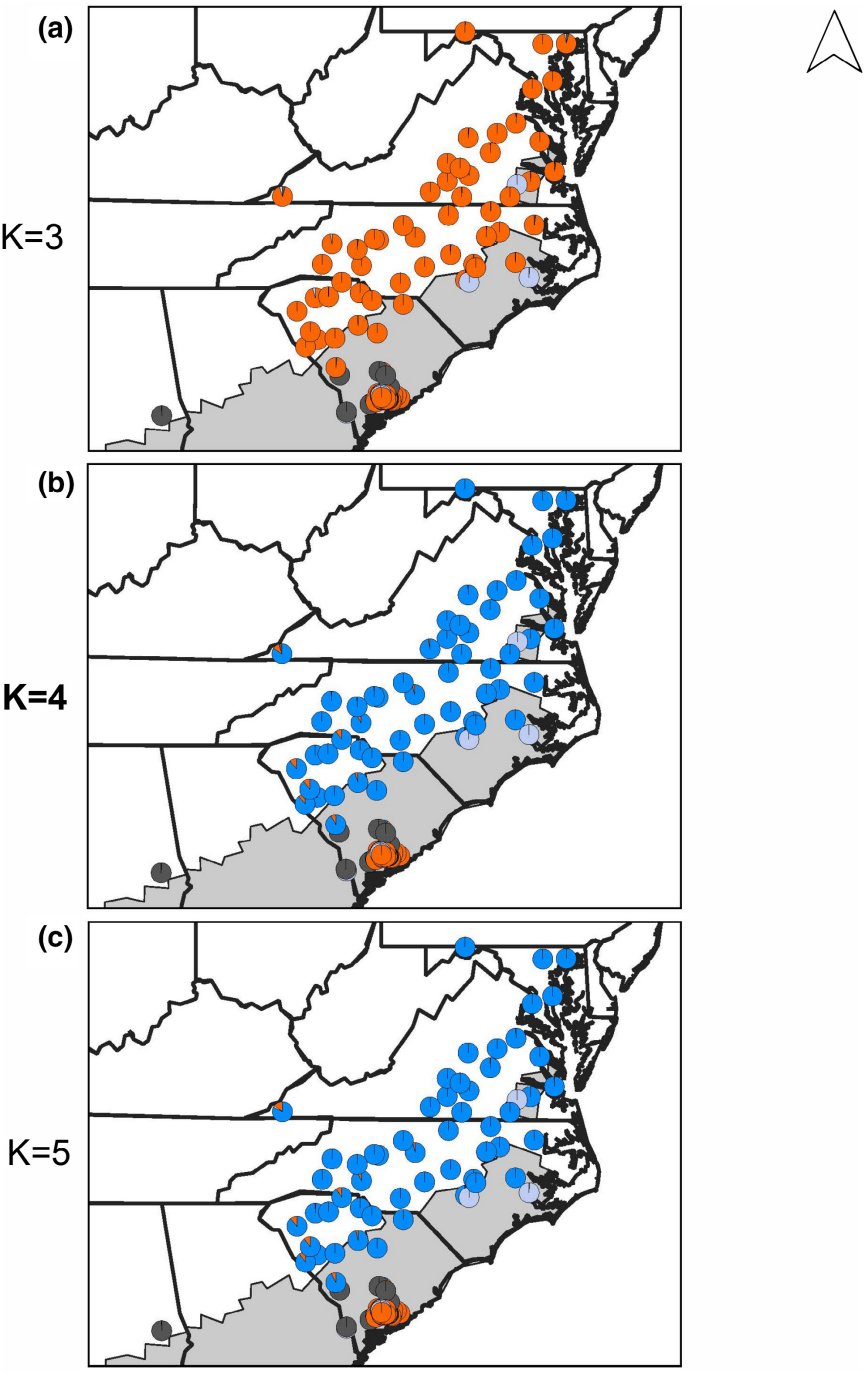
Geographic locations and admixture coefficients within the South Carolina (SC) contact zone. Analyses were conducted in fastSTRUCTURE with *P*. *feriarum*, *P*. *nigrita*, and *P*. *brimleyi* for the most likely cluster configuration (bolded, K = 4 was best‐supported; Figure S3; Table S27). Gray‐shaded area of the map represents the range of *P*. *nigrita*. For K = 3 to K = 5 (a–c), dark gray indicates sympatric and allopatric *P*. *nigrita*, and light blue gray indicates sympatric and allopatric *P*. *brimleyi*. At K = 3 (a), orange represents all *P*. *feriarum*; at higher levels of K, however, coastal sympatric populations from the Charleston, South Carolina (SC) area (orange) cluster separately from the other mainly allopatric conspecific populations (medium blue).

In South Carolina and adjacent areas (South Carolina data set), the best model of the genetic structure reflects the significant genetic divergence between sympatric *P*. *feriarum* in this region and the remainder of the species (Figure 4). Under the best model (K = 4; Table S27), sympatric and allopatric *P*. *nigrita* formed one group (dark gray), and sympatric and allopatric *P*. *brimleyi* formed a second group (light blue gray), but sympatric and allopatric *P*. *feriarum* were clustered into separate groups (orange and blue, respectively; Figure 4; Figure S3; Tables S18–S36).

**FIGURE 5:**
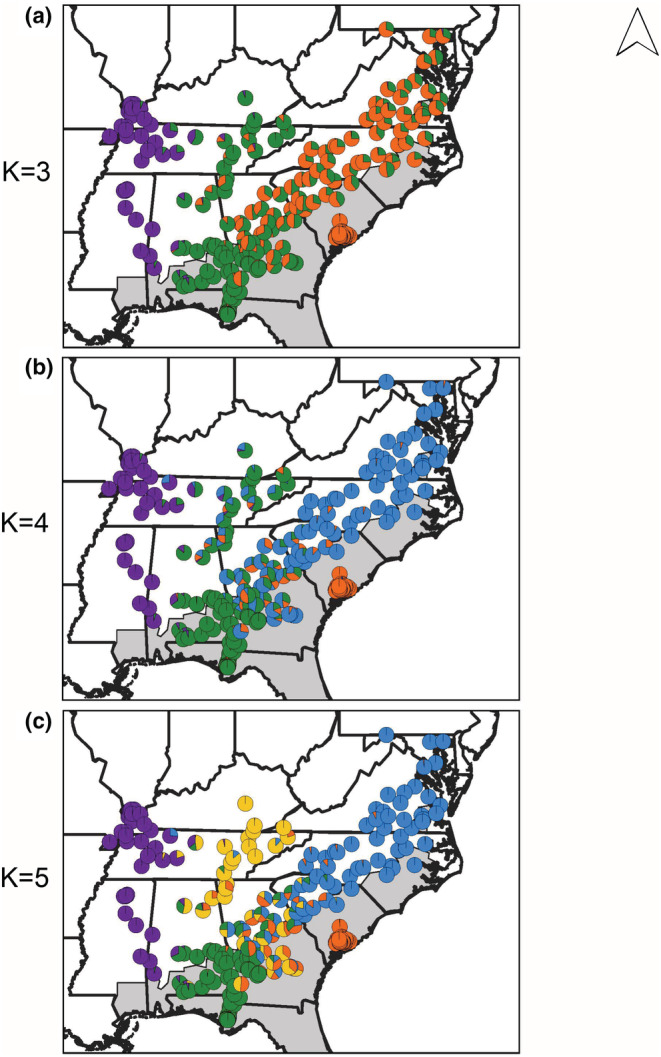
Geographic locations and admixture coefficients for all sequenced *P*. *feriarum*. Analyses were conducted in fastSTRUCTURE for the most likely cluster configurations (bolded, K = 3 and K = 5 were best‐supported; Figure S4). Gray‐shaded area of the map represents the range of *P*. *nigrita*. (a) K = 3, (b) K = 4, and (c) K = 5. For K = 5, purple indicates western allopatric populations, green indicates sympatric and allopatric populations west and south of the Appalachian mountains, orange indicates the sympatric South Carolina populations, yellow indicates an inland allopatric cluster west of the Appalachian mountains, and blue corresponds to the remaining mostly allopatric populations.

Analysis of all *P*. *feriarum* samples (Range‐wide data set) reinforces the findings from the regional analyses of multiple species above and identifies significant genetic divergence among populations across the species range. The Range‐wide *P*. *feriarum* analysis found the highest support for two models (K = 3 and K = 5; Table S27). At K = 3, the western allopatric (purple) populations, allopatric and sympatric populations from west and south of the Appalachian Mountains (green; TN, KY, AL, FL, GA,), and allopatric and sympatric populations east of the Appalachians (orange; MD, VA, NC, SC, and GA) each form a separate cluster, with admixture occurring between the latter two groups along their boundary in Georgia (Figure 5; Figure S4; Tables S4–S10). At K = 4, the same clusters are maintained, except the East‐of‐Appalachians cluster is split between a sympatric South Carolina cluster (orange) and the mostly allopatric eastern populations (blue; Figure 5). At K = 5, the West‐of‐Appalachians cluster is further divided into an inland allopatric (yellow; TN and KY) cluster, and the remaining allopatric and sympatric populations in Florida and adjacent areas (green). Under K = 5, extensive admixture has occurred along the Altamaha River drainage in central and eastern Georgia among all clusters except the western allopatric (purple) cluster (Tables S4–S10). At K = 4 and K = 5, there is little evidence of introgression into sympatric South Carolina populations (orange) from any other *P*. *feriarum* populations, suggesting strong genetic isolation in this region.

**FIGURE 6:**
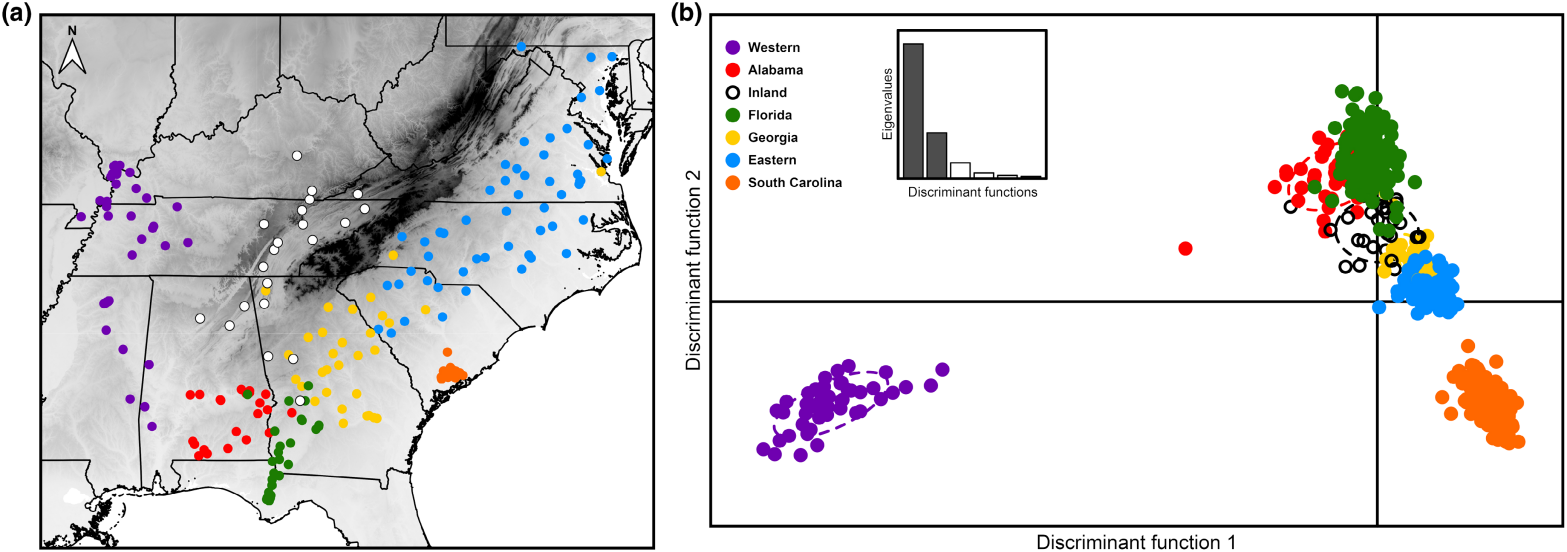
Population clusters within *P*. *feriarum* (K = 7) as defined by a discriminant analysis of principal components (DAPC). (a) Map showing the geographic position of the sampled *P*. *feriarum* individuals with colors indicating the assigned DAPC cluster. Gray‐shaded areas of the map indicate topography (darker areas correspond to higher elevation). (b) Scatter plot showing the population clusters as resulting from the first two discriminant functions (eigenvalues in the inset bar plot). DAPC clusters each include individuals from areas of sympatry with *P*. *nigrita* and/or *P*. *brimleyi*, as well as allopatric areas, except for the South Carolina (orange) and Western (purple) clusters, which include sympatric and allopatric individuals, respectively.

Estimation of population structure using DAPC concurs with the phylogenetic‐based analyses, which indicated five separate shifts of *P*. *feriarum* from allopatry into sympatry (three into the range of *P*. *nigrita* and two into the range of both *P*. *nigrita* and *P*. *brimleyi*; Banker et al., 2020; Tables S28–S30). Selection of the best cluster configuration via BIC indicated that K = 5 to K = 7 were the most likely number of clusters within the data (Figure S5). Consistent with the BIC selection, DAPC analysis recovered seven genetic clusters (Figure 6). The red, green, yellow, and blue clusters each included both allopatric and sympatric populations. This result is consistent with Banker et al. (2020), who found evidence for separate shifts into sympatry by *P*. *feriarum* by following river floodplains that bisect the Coastal Plain of the southeastern U.S. (Banker et al., 2020). The orange cluster, which includes all sympatric South Carolina samples, is genetically distinct from adjacent allopatric (blue and yellow) and other sympatric *P*. *feriarum* populations along DF2 (22.1% variance explained, Figure 6). Geographic proximity suggests that although the sympatric South Carolina cluster was probably derived from adjacent allopatric populations (Banker et al., 2020), it has since undergone sufficient divergence to be classified as a distinct genetic cluster. The western allopatric (purple) cluster was highly divergent from other *P*. *feriarum* populations along DF1 (64.8% variance explained, Figure 6).

### 3.4 Genetic structure summary statistics

Analyses of divergence and diversity among ten *P*. *feriarum* populations support the results of our clustering analyses of intraspecific genetic structure. Pairwise F_ST_ values and Nei's genetic distances reveal higher levels of differentiation between the sympatric South Carolina population and the remainder of the species, as well as between the sympatric Florida population and all other populations (Table 1, Figures S6 and S7). Tests of significance for these pairwise F_ST_ values revealed significant differentiation between every pair of populations tested (Table S31). This result is expected since the populations in some pairwise comparisons are geographically‐distant and may experience differentiation due to isolation‐by‐distance (but see Banker et al., 2020, who found significant genetic structuring within the focal species into allopatric and sympatric clusters independent of geography). D_xy_ values are very similar across population pairs, indicating that sympatric and allopatric populations within the same contact zone are as differentiated from one another as they are geographically‐distant populations (Table S32). Additionally, the sympatric South Carolina site showed relatively moderate levels of nucleotide diversity, as well as a significantly high positive value for Tajima's D (Tajima's *D* = 8.47, *p* = .013; Table 2), which indicates a lack of rare alleles potentially due to balancing selection or a population contraction (Table 2).

**TABLE 1:** Genetic divergence summary statistics for *P*. *feriarum* populations in five contact zones (AL, FL, SC, GA, VA) that are sympatric (Sym) or allopatric (Allo) with respect to *P*. *nigrita* and/or *P*. *brimleyi* (Figure 1)

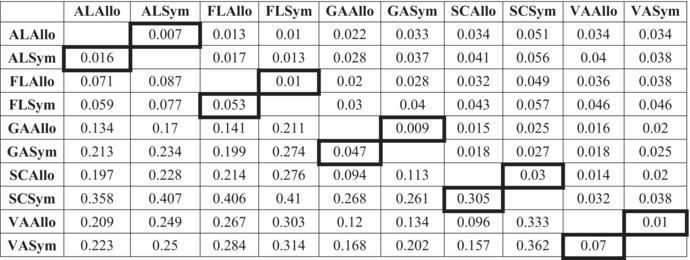

*Note*: Pairwise F_ST_ (Weir & Cockerham, 1984) values for each pair of populations are shown below the diagonal. Nei's genetic distance (Nei, 1972) values for each pair of populations are shown above the diagonal. Comparisons of sympatric and allopatric populations in the same contact zone are denoted by bold borders around the cell. Nei's genetic distance and pairwise F_ST_ values are visualized in Figures S6 and S7, respectively.

**TABLE 2:** Genetic diversity summary statistics for *P*. *feriarum* populations in five contact zones (AL, FL, SC, GA, VA) that are sympatric or allopatric with respect to *P*. *nigrita* and/or *P*. *brimleyi* (Figure 1)

Population	Mean sites	*π* (mean ± SD)	Tajima's *D*	Scaled Tajima's *D*	*p*‐Value	Rare alleles
Allopatric AL	198.2	22.12 ± 4.2615	−5.45	−0.0275	.299	Many
Sympatric AL	196	21.71 ± 4.9128	−2.58	−0.0132	.603	Many
Allopatric FL	198.3	18.7 ± 4.8364	0.65	0.0033	.888	Neutral
Sympatric FL	192.6	20.2 ± 3.6514	−0.99	−0.0051	.823	Neutral
Allopatric GA	190.9	15.94 ± 5.1085	−0.68	−0.0036	.862	Neutral
Sympatric GA	197.3	17.63 ± 4.0993	−1.17	−0.0059	.791	Neutral
Allopatric SC	201.7	17.23 ± 6.3326	0.32	0.0016	.920	Neutral
Sympatric SC	197.4	20.05 ± 4.5673	8.47	0.0429	.013*	Lacking
Allopatric VA	203.1	12.26 ± 2.5378	0.31	0.0015	.920	Neutral
Sympatric VA	200.5	19.12 ± 2.8475	4.95	0.0247	.188	Lacking

*Note*: Only the sympatric South Carolina (SC) population shows a significant *p*‐value in Tajima's *D* test of neutrality, as denoted with an asterisk. “Mean sites” refers to the mean number of variable loci in each population. Nucleotide diversity is given as pi (*π*).

### 3.5 Directionality of gene flow across contact zones

The model with the highest marginal likelihoods supported the unidirectional migration of *P*. *feriarum* alleles from allopatry into sympatry for the Altamaha (GA), Edisto (SC), and James/Anna (VA) contact zones (Table 3). For the same contact zones, the model allowing bidirectional migration was the second best‐supported model. In the case of the Escambia/Apalachicola (FL) contact zone, the model with the highest marginal likelihood indicated bidirectional migration followed by the model allowing migration only from allopatry into sympatry (Table 3). The model allowing only migration from sympatry into allopatry in the Florida contact zone did not run to completion after over 1200 CPU hours despite multiple attempts; thus this model was deemed to be unsupported by the data. Modal migration rates from allopatry to sympatry were higher in the Altamaha (GA) and James/Anna (VA) contact zones, and lower in the Edisto (SC) and Escambia/Apalachicola (FL) contact zones (Table S33).

**TABLE 3:** Directionality of gene flow across multiple hybrid zones.

Contact zone	Model	Marginal likelihood	Relative weight	Model order
Escambia/Apalachicola (FL)	Allo to Sym	−334,539.64	−1637.07	2
	**Bidirectional**	**−332,902.57**	**0**	**1**
	Sym to Allo^a^	—	—	—
Altamaha (GA)	**Allo to Sym**	**−233,656.27**	**0**	**1**
	Bidirectional	−233,895.97	−239.70	2
	Sym to Allo	−234,453.51	−797.24	3
Edisto (SC)	**Allo to Sym**	**−217,438.55**	**0**	**1**
	Bidirectional	−218,218.63	−780.08	2
	Sym to Allo	−218,680.64	−1242.09	3
James/Anna (VA)	**Allo to Sym**	**−209,558.69**	**0**	**1**
	Bidirectional	−209,569.12	−10.43	2
	Sym to Allo	−209,776.93	−218.24	3

*Note*: Models for the direction of migration were evaluated in MIGRATE‐n. For each contact zone, three different models were tested and the relative weight of their resulting marginal likelihood was used to decide the most likely scenario (model order) following Beerli and Palczewski (2010). The tested models correspond to those in Figure S1. The best‐supported model is bolded for each contact zone.

^a^ The model testing migration only from sympatry into allopatry did not finish for the AL/FL contact zone.

### 3.6 Laboratory hybrid crosses

Viable hybrid offspring can be produced through crosses between *P*. *feriarum* and *P*.*brimleyi*, although the degree of viability varied among replicate crosses. Two of four *P*. *feriarum* male × *P*. *brimleyi* female crosses produced no viable eggs (no tadpoles hatched). The third cross produced seven tadpoles from 140 eggs and the fourth produced 36 tadpoles from 60 eggs. One of the two *P*. *brimleyi* male × *P*. *feriarum* female crosses produced no viable eggs, and the other produced two tadpoles from 60 eggs. Of the three pure *P*. *brimleyi* crosses, one produced no viable eggs, the second 3 tadpoles from 80 eggs, and the third produced 33 tadpoles from 70 eggs. Of the five pure *P*. *feriarum* crosses, all produced tadpoles (*n* = 34 from 117 eggs, *n* = 9 from 120, *n* = 18 from 100, *n* = 19 from 50, and *n* = 41 from 170, respectively). In the crosses that produced no tadpoles, no amplexus was observed, but instead, the female apparently released her eggs without fertilization (Table S34). Although there was a trend toward a higher probability of tadpoles hatching in pure species (0.209) compared with hybrid crosses (0.114), the difference was not significant (test‐statistic = 0.096, *p* = .195), likely as a consequence of low statistical power due to the small number of replicate crosses (Table S34).

### 3.7 Field observations of heterospecific interactions

Field surveys confirm that *P*. *feriarum*, *P*. *nigrita*, and *P*. *brimleyi* frequently co‐occur in the same breeding ponds, with males even calling side‐by‐side with congeners at some sites, thereby providing the opportunity for hybridization. In sympatry between *P*. *feriarum* and *P*. *nigrita*, the two species were found calling together at 25 sites in Liberty Co., Florida. In sympatry among *P*. *feriarum*, *P*. *nigrita*, and *P*. *brimleyi*, the three species co‐occurred at eight sites in Colleton and Dorchester Counties., South Carolina and at 13 sites in Surrey, Sussex, and York Counties., Virginia (Table S35). At Site 31 in Colleton Co., South Carolina, a heterospecific pair (replicate 12 in the laboratory cross experiment; female *P*. *brimleyi* × male *P*. *feriarum*; Table S34) was observed in natural amplexus in the field, indicating that these species do attempt to hybridize in nature, at least sporadically. This pair was captured and allowed to continue mating in the laboratory, producing 60 eggs, 36 of which hatched; both parents were also genotyped to confirm species identity (Tables S1, S26, and S34).

### 3.8 Hybridization among the Trilling *Pseudacris*

Hybridization is widespread among members of the Trilling chorus frog clade within the genus *Pseudacris* (Table S36). Of the 10 species in this sub‐clade, 17 of 45 possible species pair combinations overlap with each other geographically to form contact zones. Of the 17 that form contact zones, 12 do show genetic evidence of hybridization, two do not, and three pairs have not been examined. The focal species of this study, *P*. *feriarum*, forms contact zones with eight other species along the periphery of its range (Lemmon, Lemmon, & Cannatella, 2007; Ospina et al., 2020). This species is known to hybridize with at least five of these congeners; for the remaining three, it does not hybridize with two and the other is undetermined (Table S36). Collectively, these data suggest that hybridization may provide a selective impetus for the behavioral diversification observed in this group.

## 4 DISCUSSION

Our work revealed that hybridization is uncommon but widespread among chorus frogs undergoing reinforcement. The focal species in our ongoing cascade reinforcement studies, *P*. *feriarum*, hybridizes with the majority of congeners that occur along the boundaries of its range; moreover, most other pairs of geographically overlapping Trilling chorus frogs also hybridize with each other occasionally. We found that hybridization is rare between the focal species and two congeners (0.7%–2%), and we did not detect advanced hybrids beyond the second generation. The focal species is structured genetically, containing multiple genetic clusters spanning contact zones with other species, with genetic sub‐structuring between reinforced and nonreinforced populations. We identified two highly divergent genetic clusters within *P*. *feriarum*, a phylogenetically‐ancestral western allopatric group and a recently‐derived sympatric South Carolina group. The latter group corresponds to populations that show strong behavioral reproductive isolation from the rest of the species (Lemmon, E. M., Ospina, O. E., Kortyna, M., Hassinger, A. B., Dye, M., Holland, S., Booker, W., Cherry, J. R., & Lemmon, A.R., unpublished data). Gene flow is generally unidirectional within *P*. *feriarum*, moving downstream from allopatry into areas of sympatry with other species in replicate river drainages. Our field observations and laboratory experiments revealed that in sympatry, *P*. *feriarum* has frequent interactions with closely‐related species at the breeding sites and is capable of producing viable hybrid offspring with these taxa. In sum, our study indicates that although *P*. *feriarum* breeds syntopically with and can generate viable offspring by mating with closely‐related taxa, hybridization now occurs infrequently.

### 4.1 Hybridization during cascade reinforcement

Here, we analyzed new genetic data for the same samples from a previous study (Lemmon & Juenger, 2017) with additional samples, a large number of nuclear loci, and very low missing data to yield high‐accuracy estimates of hybridization between *P*. *feriarum* and two congeners. With many divergent SNP markers, we have high power to accurately detect hybrids and substantially decrease the margin of error on hybridization estimates. Lemmon and Juenger (2017) detected hybridization in all sympatric populations of *P*. *feriarum* and reported the frequent occurrence of natural hybrids—31% F1 hybrids in the Florida contact zone, and 32% in Virginia—based on microsatellite markers with a high margin of error. With our more powerful nuclear data set, we detected admixed individuals between *P*. *feriarum* and *P*. *nigrita* in Florida and South Carolina at 2% and 1%, respectively (Tables S24–S26). The former estimate is consistent with Banker et al.'s (2020) identification of three admixed individuals out of 102 individuals sampled from the Florida contact zone (~3% admixed individuals). We quantified admixture between *P*. *feriarum* and *P*. *brimleyi* where they coexist in South Carolina and found a comparable level of hybridization (~1%; Tables S24–S26) as that between the former species and *P*. *nigrita*. The three species regularly co‐occur at breeding ponds (Table S35) and viable hybrids result from lab crosses of *P*. *feriarum* and *P*. *nigrita* (Lemmon, 2009; Lemmon & Lemmon, 2010) and of the former species with *P*. *brimleyi* (Table S34).

Consistent with cascade reinforcement theory (Ortiz‐Barrientos et al., 2009), our estimates of hybridization confirm a low level of admixture between *P*. *feriarum* and two congeners. Hybridization contributes to the evolution of reinforcement by generating the selection pressure that drives the divergence of reproductive behaviors between species (Abbott et al., 2013; Coyne & Orr, 2004; Howard, 1993; Servedio & Noor, 2003). Generally, ongoing hybridization during reinforcement is expected to decrease through time to a low level that maintains this selection pressure but does not erode genetic and behavioral differentiation between species (Coyne & Orr, 2004; Noor, 1999; Servedio & Kirkpatrick, 1997; Servedio & Noor, 2003). Since ongoing cascade reinforcement in *P*. *feriarum* is derived from current reinforcement in areas of sympatry with *P*. *nigrita*, our estimated prevalence of early‐generation hybrids is consistent with these theoretical predictions, as well as with empirical work in other taxa. Early‐generation hybrid adults are rare or absent in sympatric regions in systems that experience cascade reinforcement (rare: Comeault et al., 2016; Hoskin et al., 2005; Pfennig, 2003; absent: Urbanelli, 2002; Urbanelli et al., 1996) and reinforcement in the classic sense (Hopkins et al., 2012; Howard et al., 1993; Sætre et al., 1999; but also see Jiggins et al., 1997).

### 4.2 Population structure within *Pseudacris feriarum*

#### 4.2.1 Divergence between reinforced and nonreinforced populations

We found strong support for genetic divergence between sympatric and allopatric populations of *P*. *feriarum*, a predicted incidental consequence of divergence between populations undergoing cascade reinforcement (Hoskin & Higgie, 2010; Ortiz‐Barrientos et al., 2009). In Florida and adjacent populations, the *P*. *feriarum* species cluster splits into sympatric and allopatric clusters at K = 5 (Figure S2) suggesting significant differentiation between reinforced and nonreinforced populations in this contact zone. A similar pattern holds with the addition of a third congener in South Carolina. At K = 4, *P*. *nigrita* and *P*. *brimleyi* uniformly cluster within their respective species clusters, while *P*. *feriarum* splits into allopatric and sympatric clusters (Figure S3). Our summary statistics support these results since sympatric and allopatric population pairs show similar levels of absolute nucleotide divergence (D_xy_) compared with geographically‐distant population pairs (Table S32). These patterns are consistent with theoretical predictions that sympatric and allopatric populations should undergo genetic divergence when cascade reinforcement contributes to reproductive isolation (Abbott et al., 2013; Hoskin & Higgie, 2010).

Only a few other studies have tested the prediction that cascade reinforcement should generate genetic divergence between reinforced and nonreinforced conspecific populations. Hopkins et al. (2012) found only low levels of microsatellite differentiation among reinforced and nonreinforced populations of *Phlox drummondi*. Similarly, Bewick and Dyer (2014) found significant genetic differentiation between sympatric and allopatric populations of *Drosophila subquinaria* undergoing cascade reinforcement due to the presence of *D*. *recens*. A previous study utilizing a single mitochondrial marker did not find significant genetic differentiation between allopatric and sympatric populations of the former species (Jaenike et al., 2006). In another *Drosophila* species pair (*D*. *yakuba* and *D*. *santomea*), interpopulation crosses (one allopatric and one sympatric parent) result in fewer viable offspring, which prevents alleles for coevolved, sympatric reproductive traits from spreading outside the contact zone (Comeault et al., 2016). These and few other studies suggest that selection for traits that reduce maladaptive hybridization between species may also drive genetic differentiation within species (Pfennig & Rice, 2014; Rice & Pfennig, 2010; Urbanelli, 2002).

#### 4.2.2 Genetically‐differentiated populations

Two groups of populations emerge in the Range‐wide *P*. *feriarum* analyses as genetically isolated and distinct from the remainder of the species. At K = 2, there is a general split by geography into clusters representing populations from East‐ and West‐of‐the‐Appalachians (Figure S4; Tables S4–S10). However, the better‐supported models (K = 3 and K = 5; Figure S5; Table S27) also reveal two highly distinct genetic clusters—a western allopatric group and a behaviorally‐distinct sympatric South Carolina group—and these groups show low connectivity to the rest of the species. These results are robust to the scale of the data set (regional interspecies or Range‐wide intraspecies) and type of analysis (fastSTRUCTURE, DAPC, genetic diversity, and divergence summary statistics; Figures 5 and 6, Tables 1 and 2, Figures S2–S4 and S6, S7, Tables S4–S10, S28–S30, and S32). Consistent with previous works that were limited in either statistical power or geographic scale (Banker et al., 2020; Lemmon & Juenger, 2017), we found that the sympatric South Carolina and western allopatric clusters behave as mostly or fully reproductively‐isolated units, respectively, relative to the whole of the *P*. *feriarum* species. The sympatric South Carolina population also shows a significantly high value of Tajima's D (Tajima's D = 8.47, *p* = .013; Table 2), which indicates a lack of rare alleles, possibly driven by balancing selection. Males from the sympatric South Carolina populations produce a distinct advertisement call, and females show strong preferences for male signals from their own local population (Lemmon, E. M., Ospina, O. E., Kortyna, M., Hassinger, A. B., Dye, M., Holland, S., Booker, W., Cherry, J. R., & Lemmon, A.R., unpublished data). Future work will investigate the barriers to gene flow in the western allopatric cluster as well.

### 4.3 Directionality of gene flow across contact zones

Contrary to some theoretical models, we found evidence of directional gene flow from allopatry into sympatry in multiple contact zones. Our data are best‐supported by this model of unidirectional gene flow in three of the four contact zones included in our analysis (Table 3). There are at least three main explanations for the results of our gene flow directionality analyses, including (1) strong sexual selection against hybrids in sympatry, (2) low gene flow, and (3) a flood model of gene migration (Jacquemyn et al., 2006).

First, theoretical models reveal that reinforcement in the face of gene flow is not only possible (Liou & Price, 1994) but also that even the extreme case of unidirectional gene flow into sympatry (i.e., from a continent to an island) does not preclude reinforcement (Servedio & Kirkpatrick, 1997). These authors argue that bidirectional gene flow eases the evolution of reinforcement since preference alleles may migrate back into a population after flowing out several generations prior (Servedio & Kirkpatrick, 1997). Empirical support for the evolution of reinforcement under bidirectional gene flow exists in *Ficedula* flycatchers (Sætre et al., 1999) and *Timema* stick insects (Nosil et al., 2003, 2007). A later theoretical model with less restrictive assumptions, however, suggested that reproductive isolation may evolve more easily under unidirectional gene flow if signaling traits differ among populations, and genetically‐variable preferences act upon this difference (Kirkpatrick & Servedio, 1999). Therefore, it is possible that the directionality of gene flow detected in *P*. *feriarum* is a consequence of strong sexual selection in this system, but this condition is not necessary to explain the persistence of reinforced traits in sympatry.

A second, and more likely, explanation for the directionality of gene flow in this system is that the rate of gene flow remains insufficient to weaken genetic linkages that generate isolating barriers. The unidirectional gene flow we observed is consistent with the predictions of a neural network model of cascade reinforcement in which reproductive isolation is maintained only when gene flow from sympatry into allopatry is minimal (Yukilevich & Aoki, 2016). The same authors also found that gene flow from allopatry into sympatry has no effect on the maintenance of reproductive isolation, so long as the gene flow remains below the threshold at which the epistatic linkages between traits and preferences recombine (Yukilevich & Aoki, 2016). Further empirical support for cascade reinforcement in the face of directional gene flow exists in Morning glory flowers (*Ipomoea*), where gene flow occurs asymmetrically from one species (*I*. *lacunosa*) to a sympatric congener (*I*. *cordatotriloba*). Crucially, this asymmetry in interspecific gene flow contributes to the enhancement of reproductive barriers both between sympatric *I*. *lacunosa* and its sympatric congener and allopatric conspecifics (Ostevik et al., 2020). However, the existence of genetic linkages that contribute to mate recognition in *P*. *feriarum* remains unknown. In other reinforced systems, workers have identified the genetic mechanisms of reinforcement, including candidate gene sets related to assortative mating (Smadja et al., 2015), one‐allele mechanisms (Bousquet et al., 2012; Marcillac et al., 2005; Ortíz‐Barrientos & Noor, 2005), and coupled genetic mechanisms for mate recognition (Sætre et al., 2003; Xu & Shaw, 2019). Further studies may include high‐resolution genetic analyses of *P*. *feriarum* call characteristics and female preferences to determine the genetic mechanisms that contribute to mate recognition in this system, which may then clarify the effect of the relative strength of gene flow on the population genetic landscape.

Our findings lend support to a third alternative explanation for directional gene flow from allopatry into sympatry in this system—the flood model of gene migration in *P*. *feriarum* (as proposed by Jacquemyn et al., 2006). In each pair of populations that we selected to represent one contact zone, sympatric populations are positioned downstream from allopatric populations along river drainages (Figure S1; Banker et al., 2020). Cross‐river downstream populations share more genetic similarities than cross‐river upstream populations, therefore it is likely that animals are washed downstream from inland, allopatric populations during seasonal storms (Michelsohn, 2012). Our results further support Michelsohn's (2012) flood model of gene migration in this system, resulting in one‐way gene flow from upstream allopatric populations to downstream sympatric populations. While our analyses of gene flow focus on the directionality of flow between sympatric and allopatric populations of *P*. *feriarum*, future work should explicitly test the prediction of cascade reinforcement that gene flow rate should be low between sympatry and allopatry (modal migration rates recovered from our analyses are included in Table S33).

### 4.4 Multi‐species interactions and the potential for species diversification

#### 4.4.1 Hybridization with congeneric taxa at range periphery

Hybridization between *P*. *feriarum* and at least five congeneric species at the periphery of its range sets the stage for rapid species proliferation by cascade reinforcement within the focal species (interactions with *P*. *nigrita*, *P*. *triseriata*, *P*. *brimleyi*, *P*. *brachyphona*, and *P*. *collinsorum*; Table S36). Each of these heterospecific taxa differs in mating call phenotype from *P*. *feriarum*, varying in pulse rate and pulse number, which are the most salient features of the signal during mate choice (Lemmon, 2009; Lemmon & Lemmon, 2010). Because the direction and magnitude of signal divergence in *P*. *feriarum* appear to be influenced by the signals of heterospecifics present in their community (Lemmon, E. M., Ospina, O. E., Kortyna, M., Hassinger, A. B., Dye, M., Holland, S., Booker, W., Cherry, J. R., & Lemmon, A.R., unpublished data; Lemmon, 2009), these species are likely to contribute substantially to the diversification of *P*. *feriarum* populations. In fruit flies experiencing cascade reinforcement, reproductive isolation between *D*. *subquinaria* and the sympatric *D*. *recens* has the incidental effect of increasing isolation between the former species and *D*. *transversa* (allopatric with respect to *D*. *subquinaria*, but sympatric with *D*. *recens*; Humphreys et al., 2016). Given these findings, we might predict that sympatric *P*. *feriarum* would not only discriminate against local heterospecifics and allopatric conspecifics but also allopatric heterospecifics. Indeed, Lemmon, E. M., Ospina, O. E., Kortyna, M., Hassinger, A. B., Dye, M., Holland, S., Booker, W., Cherry, J. R., & Lemmon, A.R., unpublished data) in silico estimates demonstrate that reproductive isolation with respect to other species increases during cascade reinforcement. This prediction is consistent with work involving two‐species interactions that drive cascade reinforcement in killifish, where sympatric females discriminate against both heterospecific males and foreign conspecific males (Kozak et al., 2015). However, in another two‐species system experiencing cascade reinforcement, sympatric females discriminate against allopatric conspecifics but not against foreign sympatric conspecifics (Porretta & Urbanelli, 2012). Ongoing work will investigate the evidence for cascade reinforcement at other species boundaries.

#### 4.4.2 Consequences of a three‐species hybrid zone

Theory predicts that a rapid species radiation can result from cascade reinforcement when conspecific populations experience different heterospecific assemblages (Calabrese & Pfennig, 2020; McPeek & Gavrilets, 2006; Pfennig & Ryan, 2007). Furthermore, communities that are more species‐rich have the potential to generate selection pressures that contribute to the evolution of extreme behavioral phenotypes, that differ from the remainder of the species (Calabrese & Pfennig, 2020). Here, we find an example of this situation in the sympatric South Carolina *P*. *feriarum*. Indeed, at this site, interactions with *P*. *nigrita* and *P*. *brimleyi* have driven *P*. *feriarum* mating signals to displace in pulse number, but not pulse rate, in contrast to other contact zones with *P*. *nigrita* alone (Lemmon, 2009). Crucially, sympatric South Carolina females are choosier than both allopatric females and sympatric females from other localities (Lemmon, E. M., Ospina, O. E., Kortyna, M., Hassinger, A. B., Dye, M., Holland, S., Booker, W., Cherry, J. R., & Lemmon, A.R., unpublished data). Therefore, to our knowledge, this population represents the only recorded instance of two species with different signal characteristics (*P*. *nigrita* and *P*. *brimleyi*) contributing to behavioral and genetic divergence of a reinforced population of a third species (*P*. *feriarum*).

### 4.5 Limitations

There are three main limitations of this study. First, we cannot discount the impacts of environmental variation on diversification and the evolution of reproductive isolation in this clade (Barton, 2013; Doebeli & Dieckmann, 2000; Gavrilets, 2004; Rundle & Nosil, 2005; Schluter, 2001; Servedio et al., 2013). Unfavorable or patchy habitat at the periphery of a species range could contribute to geographic isolation that gives rise to genetically‐divergent populations (peripatric speciation; Mayr, 1954; Coyne & Orr, 2004; empirically demonstrated by Carson & Kaneshiro, 1976; Givnish et al., 2009; Lewis, 1973; Shaw, 2002). In our study, the focal species has shifted into novel riverine floodplain environments in sympatric areas that may provide these conditions for divergence. Furthermore, frogs in each contact zone likely experience different selection pressures from the many other environmental factors that can contribute to reproductive isolation (Mandeville et al., 2015; Nosil et al., 2007; Papa et al., 2008; Rice et al., 2016). Malone et al. (2014) tested for environmental selection on male calling traits in allopatric and sympatric *P*. *feriarum* populations and found no evidence for this process. Evidence that reproductive isolation between sympatry and allopatry exists and is attributable to reinforcement, rather than differences in the local environment alone, would be strengthened by reciprocal crossing experiments between sympatric and allopatric populations. If the degree of trait divergence between a pair of sympatric and allopatric populations predicts the degree of reproductive isolation between those two populations (as measured by reproductive success in a testcross), then reinforced traits are likely to reproductive isolation (Pfennig, 2016). Although the role of geography cannot be ruled out, the preponderance of evidence in this system suggests that the key factor driving speciation is the repeated reinforcement of mating behaviors (Lemmon, E. M., Ospina, O. E., Kortyna, M., Hassinger, A. B., Dye, M., Holland, S., Booker, W., Cherry, J. R., & Lemmon, A.R., unpublished data; Banker et al., 2020; Lemmon & Juenger, 2017). A second limitation of this study is the potential for the false discovery of recent admixture due to strong genetic drift or other deviations from the underlying assumptions of the genetic clustering algorithm we applied (Lawson et al., 2018). Although we cannot rule out the possibility of “ghost admixture” or recent population bottlenecks in some populations as outlined by Lawson et al. (2018), our identification of admixed individuals in nature is validated by the inclusion of lab‐generated hybrid animals in this analysis. A third limitation is the low number of laboratory hybrid crosses between *P*. *feriarum* and *P*. *brimleyi*, which was limited due to logistical difficulties. From the present data, we cannot conclude whether these hybrids differ in viability from pure‐species offspring, and we lack data entirely on the fitness of adult hybrids in terms of mating success. Even after we increase our replication, if hybrids show no difference in viability, further work must be done to determine whether hybrids suffer low fertility due to their less attractive, intermediate call traits, as found in *P*. *feriarum‐P*. *nigrita* hybrids (Lemmon & Lemmon, 2010). In future studies, we will assess the cost of hybridization with a larger sample across the life cycle.

### 4.6 Summary

In this study, we characterized population genetic patterns associated with cascade reinforcement and tested key predictions deriving from this process. We found that gene flow is typically unidirectional from allopatry into sympatry in a species undergoing cascade reinforcement, as predicted by some theoretical models. We determined that genetic differentiation has developed between reinforced and nonreinforced populations, which is also consistent with the theory. Our genetic survey of the entire range of the focal species revealed the presence of cryptic, highly divergent genetic clusters, that may represent incipient species. Furthermore, our admixture analyses, literature review, and examination of unpublished data indicated that the focal species hybridizes at a low frequency with at least five closely‐related congeners at the periphery of its range. In sum, this study provides the first insight into species‐wide patterns of population genetic differentiation during cascade reinforcement and lends considerable support to predictions from theory. The unique geographic and genetic framework of the study system we describe here provides an ideal opportunity for further studies to test how complex species interactions can promote the proliferation of new species.

## AUTHOR CONTRIBUTIONS

**Carlie B. Anderson:** Conceptualization (supporting); project administration (equal); supervision (equal); writing – original draft (lead); writing – review and editing (equal). **Oscar Ospina:** Data curation (equal); formal analysis (lead); validation (equal); visualization (lead); writing – review and editing (supporting). **Peter Beerli:** Formal analysis (supporting); methodology (equal); software (equal); writing – review and editing (supporting). **Alan Lemmon:** Data curation (equal); formal analysis (equal); methodology (equal); software (equal); writing – review and editing (supporting). **Sarah E Banker:** Investigation (equal); resources (supporting); writing – review and editing (supporting). **Alyssa Bigelow Hassinger:** Investigation (equal); resources (supporting); writing – review and editing (supporting). **Mysia Dye:** Formal analysis (supporting); resources (equal). **Michelle L Kortyna:** Investigation (equal); resources (lead). **Emily Claire Moriarty Lemmon:** Conceptualization (lead); funding acquisition (lead); project administration (equal); supervision (equal); writing – original draft (supporting); writing – review and editing (equal).

## CONFLICT OF INTEREST

The authors declare no competing interests.

## Supporting information


Figure S1.

Figure S2.

Figure S3.

Figure S4.

Figure S5.

Figure S6.

Figure S7.

Tables S1–S36.

## Data Availability

All supplemental materials, MIGRATE‐n parameter files, and the SNP data set are archived at https://doi.org/10.5061/dryad.r4xgxd2hm.
