# Supplementary material for: The population genetics of speciation by cascade reinforcement
Source: Ecol Evol. 2023 Feb 7;13(2):e9773. doi: 10.1002/ece3.9773 (PMC9905665; doi:10.1002/ece3.9773)
Supplement: Supplementary file 1 — Figure S1. [file ECE3-13-e9773-s008.pdf]

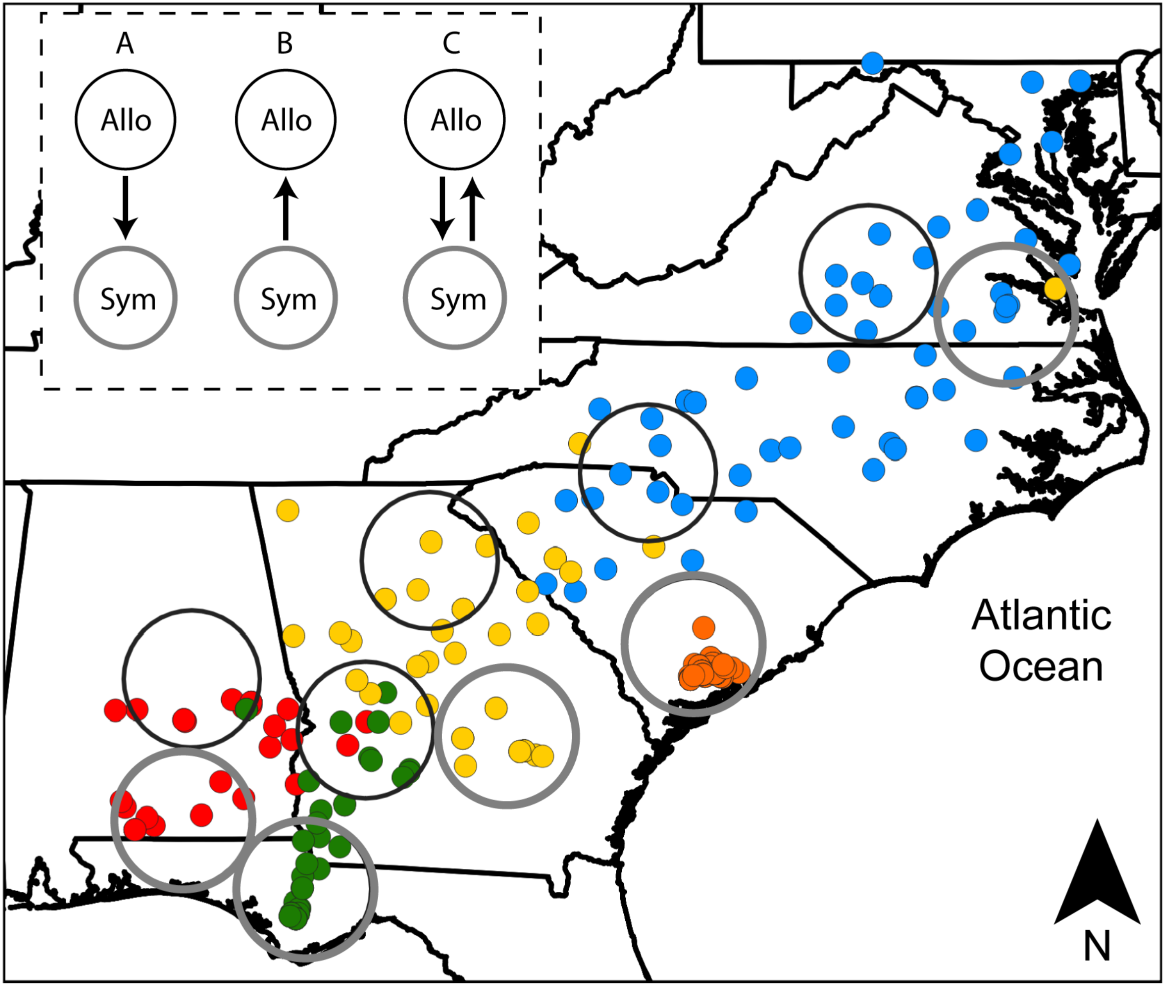

Supplemental Figure 1.Geographic locations of the *P. feriarum* samples used in the MIGRATE-n models. Colors correspond to the different DAPC clusters in Figure 6. Circles represent 150km diameter, which were used as a guide to subsample no more than 20 individuals per population (black circles = allopatry and gray circles = sympatry). Inset: Three models of gene flow were tested for each pair of subsampled DAPC clusters, A) unidirectional migration only from allopatry to sympatry, B) unidirectional migration only from sympatry to allopatry, and C) bidirectional migration.
