# Supplementary material for: The population genetics of speciation by cascade reinforcement
Source: Ecol Evol. 2023 Feb 7;13(2):e9773. doi: 10.1002/ece3.9773 (PMC9905665; doi:10.1002/ece3.9773)
Supplement: Supplementary file 2 — Figure S2. [file ECE3-13-e9773-s001.pdf]

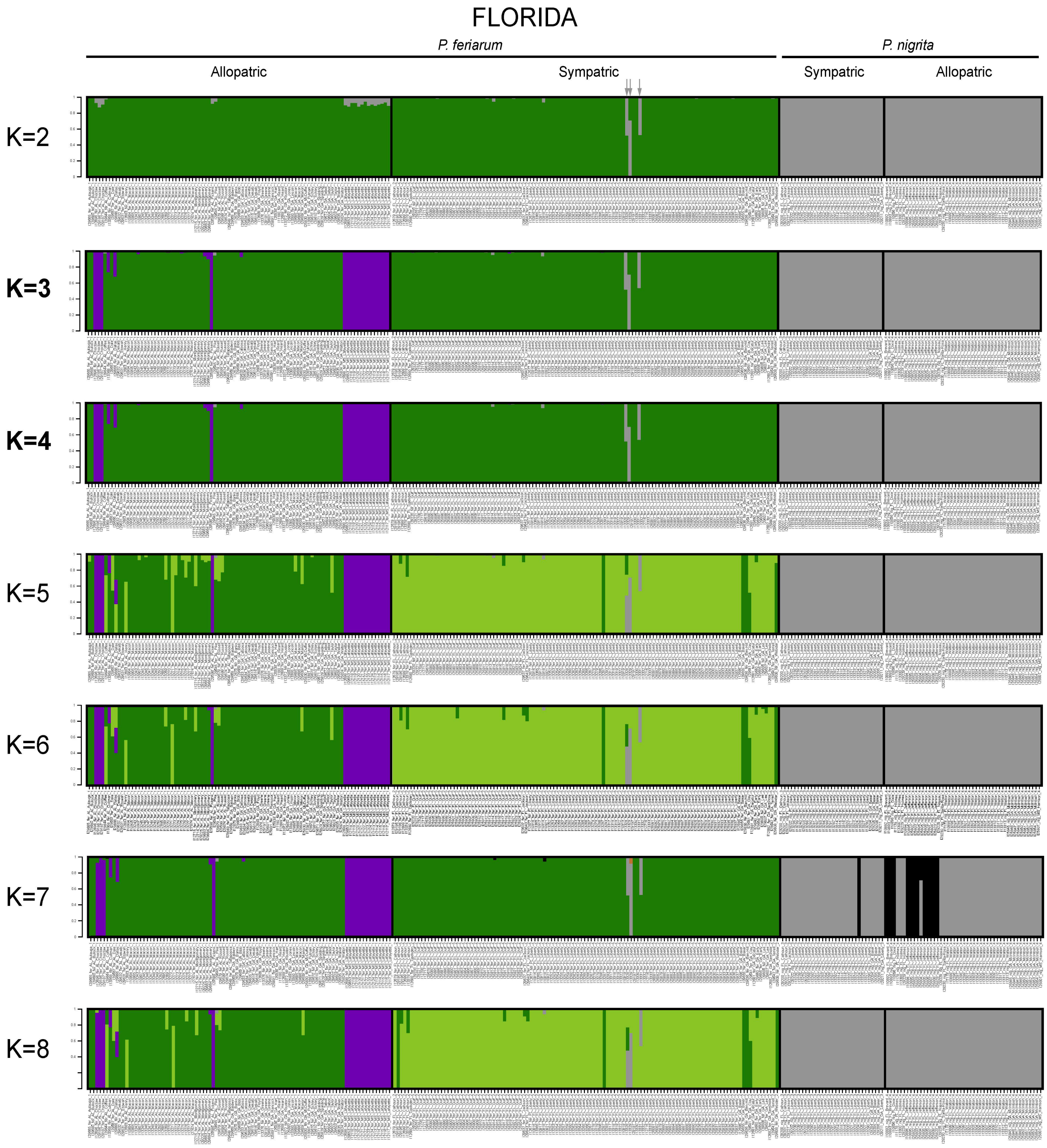

Supplemental Figure 2. Admixture coefficients estimated in the Florida (FL) contact zone for *P. feriarum* and *P. nigrita*. Analyses were performed in fastSTRUCTURE assuming different cluster configurations (K=2 to K=8). Each vertical bar represents an individual sample, with colors showing assignments to a population. Bolded Ks in the left margin indicate the most likely configurations. The small gray arrows at the top indicate putative hybrids between the two species. At K=3 and K=4, dark gray indicates sympatric and allopatric *P. nigrita*, green indicates sympatric and most allopatric *P. feriarum*, purple indicates a western allopatric *P. feriarum* cluster. At higher K-values, dark green indicates a second allopatric *P.feriarum* cluster and lime green indicates a sympatric *P. feriarum* cluster.
