# Supplementary material for: The population genetics of speciation by cascade reinforcement
Source: Ecol Evol. 2023 Feb 7;13(2):e9773. doi: 10.1002/ece3.9773 (PMC9905665; doi:10.1002/ece3.9773)
Supplement: Supplementary file 3 — Figure S3. [file ECE3-13-e9773-s007.pdf]

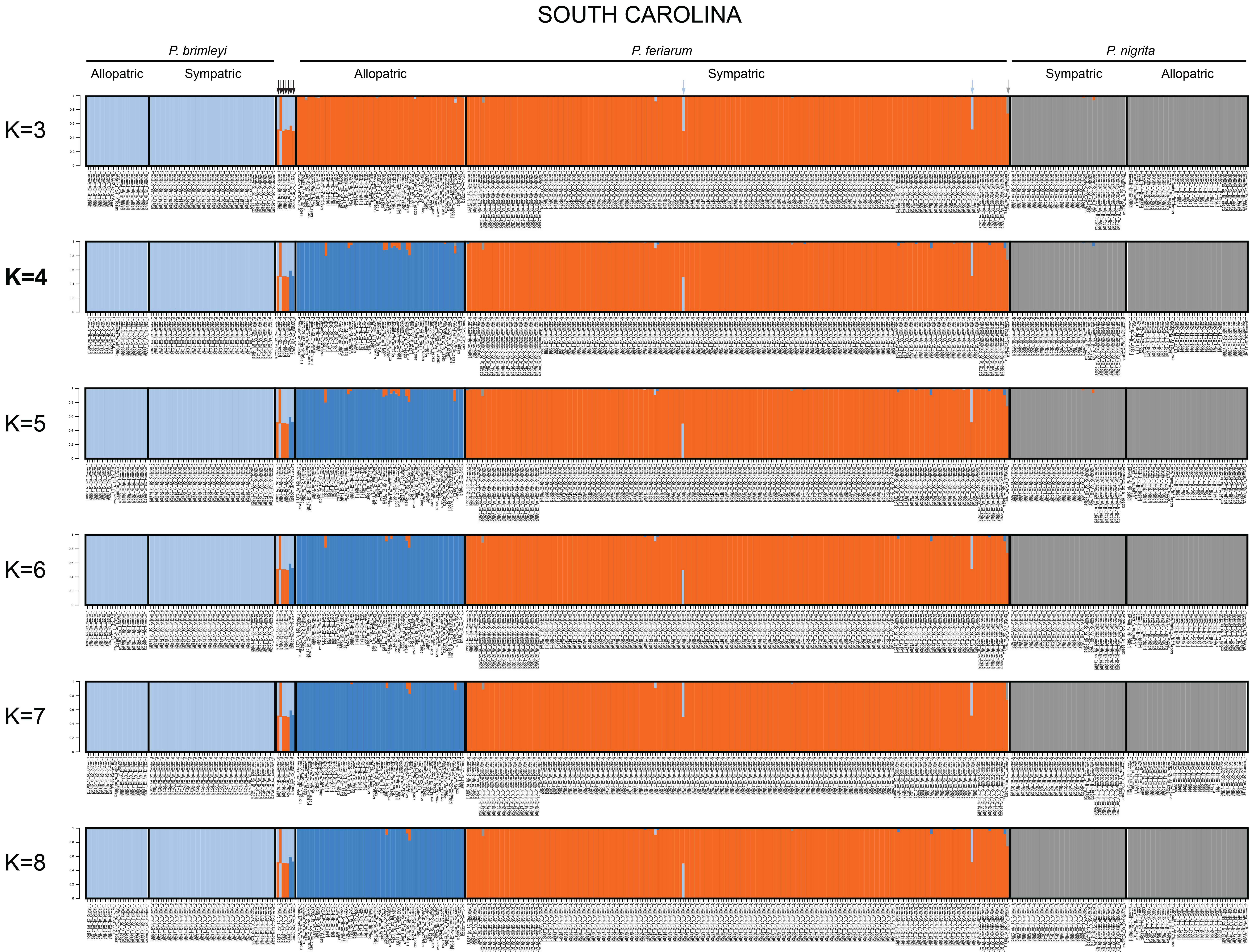

Supplemental Figure 3. Admixture coefficients estimated in the South Carolina (SC) contact zone for *P. feriarum, P. nigrita,* and *P. brimleyi.* Analyses were performed in fastSTRUCTURE assuming different cluster configurations (K=3 to K=8). Each vertical bar represents an individual sample, with colors showing assignments to a population. Bolded Ks in the left margin indicate the most likely configurations. The small gray arrow at the top indicates putative hybrids between *P. feriarum* and *P. nigrita.* The two light blue arrows indicate natural hybrids detected in the wild between *P. feriarum* and *P. brimleyi.* The black arrows indicate lab-generated hybrids between *P. feriarum* and *P. brimleyi.* The bolded Ks in the left margin indicate the most likely configurations. At K=4, dark gray indicates sympatric and allopatric *P. nigrita,* light blue gray indicates sympatric and allopatric *P. brimleyi,* orange indicates sympatric *P. feriarum*, and blue indicates allopatric *P. feriarum.*
