# Supplementary material for: The population genetics of speciation by cascade reinforcement
Source: Ecol Evol. 2023 Feb 7;13(2):e9773. doi: 10.1002/ece3.9773 (PMC9905665; doi:10.1002/ece3.9773)
Supplement: Supplementary file 4 — Figure S4. [file ECE3-13-e9773-s003.pdf]

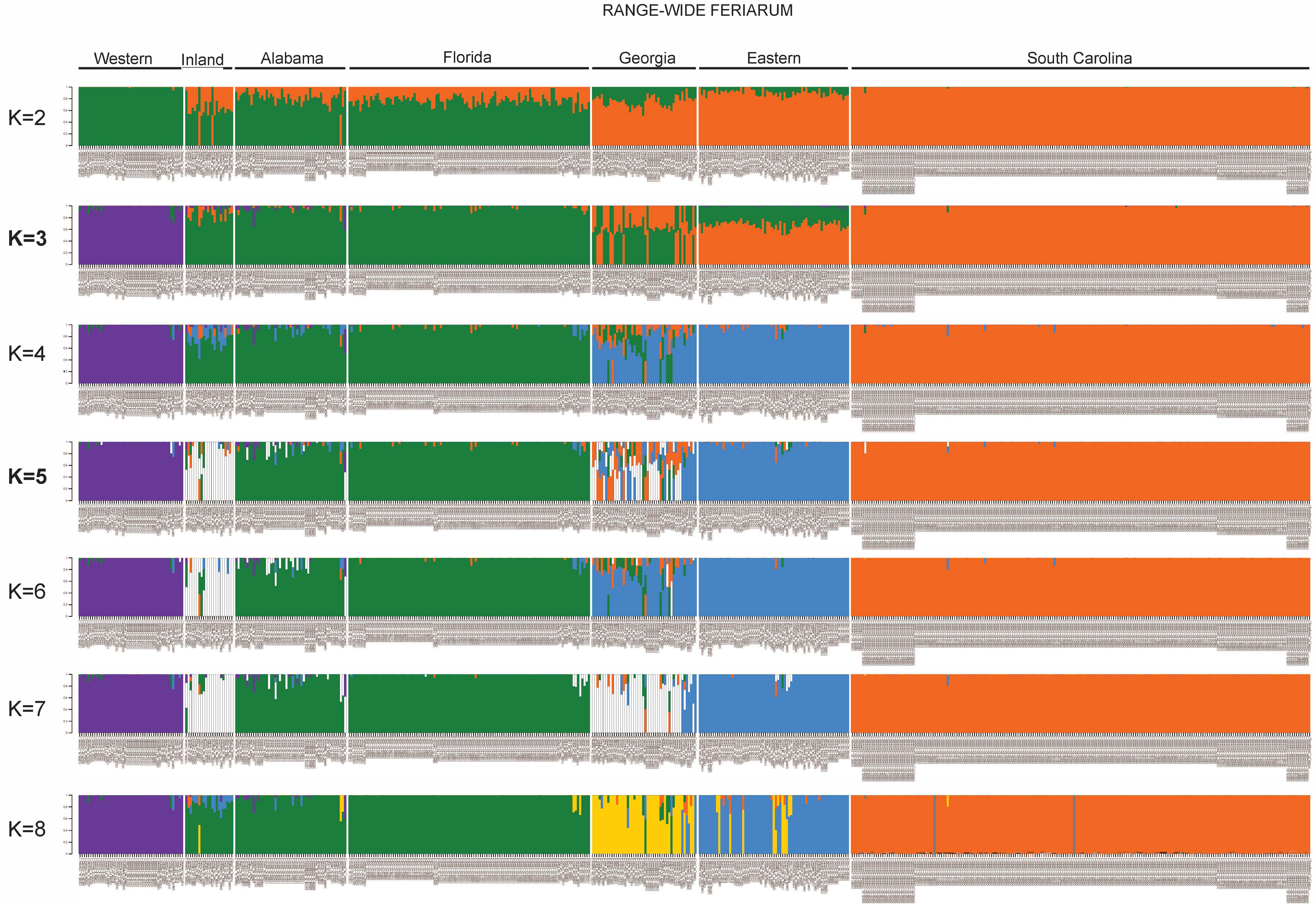

Supplemental Figure 4. Admixture coefficients estimated for all sequenced *P. feriarum*. Analyses were performed in fastSTRUCTURE assuming different cluster configurations (K=2 to K=8). Each vertical bar represents an individual sample, with colors showing assignments to a population. Bolded Ks in the left margin indicate the most likely configurations. At K=5, purple indicates western allopatric populations, green indicates sympatric and allopatric populations west and south of the Appalachian mountains, orange indicates the sympatric South Carolina populations, white indicates an inland allopatric cluster west of the Appalachian mountains, and blue indicates the remaining mostly allopatric eastern populations. Note that the Inland cluster is represented by yellow, rather than white in Figure 6.
