# Supplementary material for: The population genetics of speciation by cascade reinforcement
Source: Ecol Evol. 2023 Feb 7;13(2):e9773. doi: 10.1002/ece3.9773 (PMC9905665; doi:10.1002/ece3.9773)
Supplement: Supplementary file 5 — Figure S5. [file ECE3-13-e9773-s005.pdf]

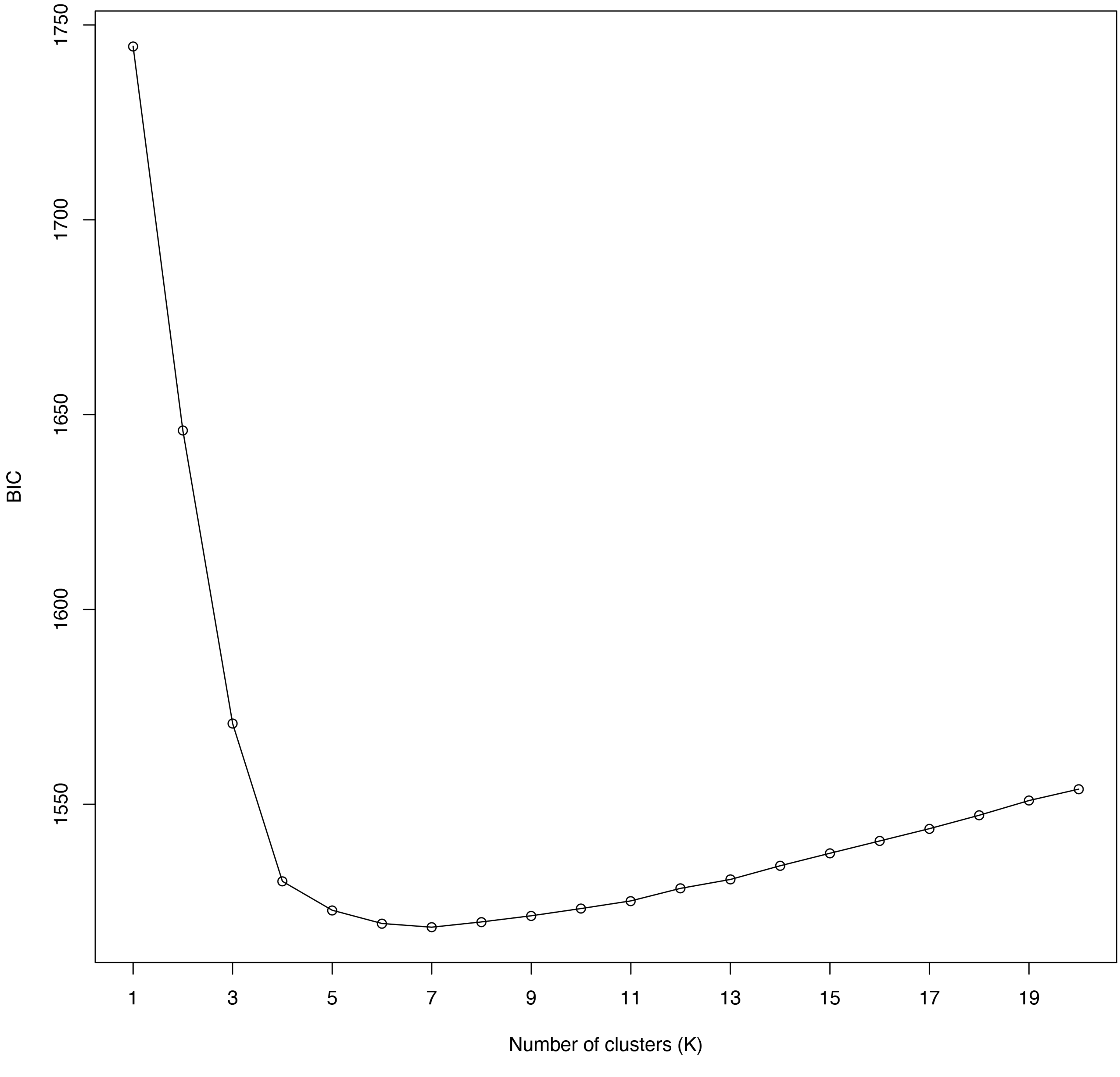

Supplemental Figure 5. Bayesian Information Criteria (BIC) for each of seven cluster configurations (K=2 to K=8) for a range-wide *P. feriarum* Discriminant Analysis of Principal Components (DAPC) analysis. K=5 to K=7 are the most likely number of clusters in the data.
