# Supplementary material for: The population genetics of speciation by cascade reinforcement
Source: Ecol Evol. 2023 Feb 7;13(2):e9773. doi: 10.1002/ece3.9773 (PMC9905665; doi:10.1002/ece3.9773)
Supplement: Supplementary file 6 — Figure S6. [file ECE3-13-e9773-s002.pdf]

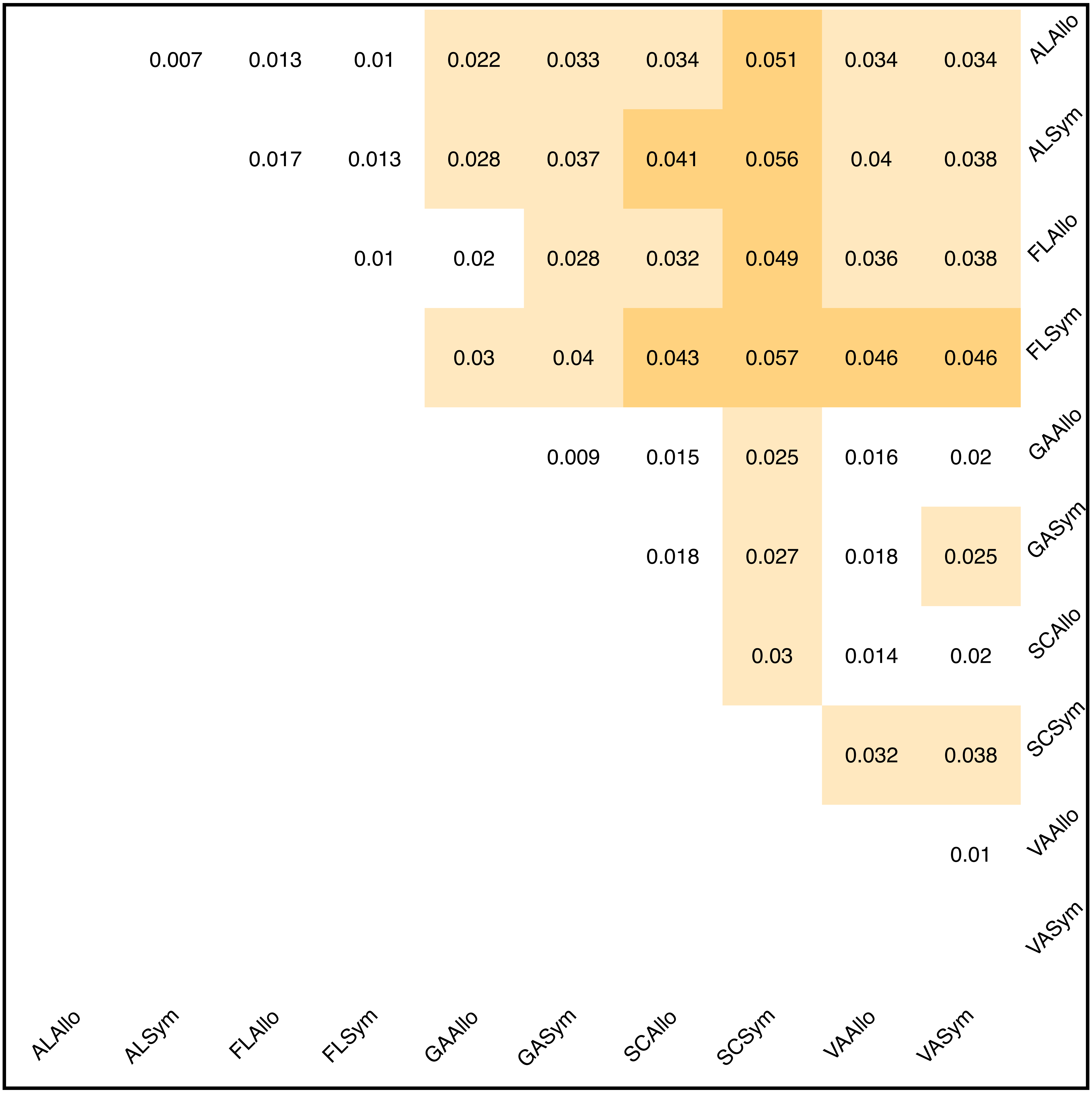

Supplemental Figure 6. Nei's genetic distance values (Nei, 1972) for each pair of *P. feriarum* populations in five contact zones (AL, FL, SC, GA, VA) that are sympatric (Sym) or allopatric (Allo) with respect to *P. nigrita* and/or *P. brimleyi*. More saturated hues indicate greater genetic distances. Genetic distance values are shown in Table 1 in the main text.
